# GATA-3 is a proto-oncogene in T-cell lymphoproliferative neoplasms

**DOI:** 10.1038/s41408-022-00745-y

**Published:** 2022-11-04

**Authors:** Xiangrong Geng, Chenguang Wang, Xin Gao, Pinki Chowdhury, Jonathan Weiss, José A. Villegas, Badeia Saed, Thilini Perera, Ying Hu, John Reneau, Maria Sverdlov, Ashley Wolfe, Noah Brown, Paul Harms, Nathanael G. Bailey, Kedar Inamdar, Alexandra C. Hristov, Trilokraj Tejasvi, Jaime Montes, Carlos Barrionuevo, Luis Taxa, Sandro Casavilca, J. Luís Alberto de Pádua Covas Lage, Hebert Fabrício Culler, Juliana Pereira, John S. Runge, Tingting Qin, Lam C. Tsoi, Hanna S. Hong, Li Zhang, Costas A. Lyssiotis, Rintaro Ohe, Tomomi Toubai, Alejandro Zevallos-Morales, Carlos Murga-Zamalloa, Ryan A. Wilcox

**Affiliations:** 1 Department of Internal Medicine, Division of Hematology and Oncology, University of Michigan, Ann Arbor, MI USA; 2 Department of Pediatrics, Dayton Children’s Hospital, Wright State University Boonshoft School of Medicine, Dayton, OH USA; 3 Department of Pharmaceutical Sciences, College of Pharmacy, University of Illinois at Chicago, Chicago, IL USA; 4 Department of Chemistry, College of Liberal Arts and Sciences, University of Illinois Chicago, Chicago, IL USA; 5 Department of Medicine, Division of Hematology, The Ohio State University Comprehensive Cancer Center, Columbus, OH USA; 6 Department of Pathology, University of Illinois Chicago, Chicago, IL USA; 7 Department of Pathology, University of Michigan, Ann Arbor, MI USA; 8 Division of Hematopathology, University of Pittsburgh, Pittsburgh, PA USA; 9 Department of Pathology, Henry Ford Hospital, Detroit, MI USA; 10 Department of Dermatology, University of Michigan, Ann Arbor, MI USA; 11 Department of Pathology, Instituto Nacional de Enfermedades Neoplásicas (INEN), Lima, Peru; 12 Department of Hematology, Hemotherapy and Cell Therapy, Faculty of Medicine, Sao Paulo University, Laboratory of Medical Investigation 31 in Pathogenesis and Directed Therapy in Onco-Immuno-Hematology, Sao Paulo, Brazil; 13 Department of Hematology, Hemotherapy and Cell Therapy, Faculty of Medicine, Sao Paulo University, Non-Hodgkin’s Lymphomas and Histiocytic Disorders, Sao Paulo, Brazil; 14 Michigan Medicine, University of Michigan, Ann Arbor, MI USA; 15 Department of Computational Medicine and Bioinformatics, University of Michigan, Ann Arbor, MI USA; 16 Department of Molecular and Integrative Physiology, University of Michigan, Ann Arbor, MI USA; 17 Department of Pathology, Faculty of Medicine, Yamagata University, Yamagata, Japan; 18 Department of Internal Medicine III, Division of Hematology and Cell Therapy, Yamagata University of Medicine, Yamagata, Japan

**Keywords:** Oncogenes, Oncogenesis

## Abstract

Neoplasms originating from thymic T-cell progenitors and post-thymic mature T-cell subsets account for a minority of lymphoproliferative neoplasms. These T-cell derived neoplasms, while molecularly and genetically heterogeneous, exploit transcription factors and signaling pathways that are critically important in normal T-cell biology, including those implicated in antigen-, costimulatory-, and cytokine-receptor signaling. The transcription factor GATA-3 regulates the growth and proliferation of both immature and mature T cells and has recently been implicated in T-cell neoplasms, including the most common mature T-cell lymphoma observed in much of the Western world. Here we show that GATA-3 is a proto-oncogene across the spectrum of T-cell neoplasms, including those derived from T-cell progenitors and their mature progeny, and further define the transcriptional programs that are GATA-3 dependent, which include therapeutically targetable gene products. The discovery that p300-dependent acetylation regulates GATA-3 mediated transcription by attenuating DNA binding has novel therapeutic implications. As most patients afflicted with GATA-3 driven T-cell neoplasms will succumb to their disease within a few years of diagnosis, these findings suggest opportunities to improve outcomes for these patients.

## Introduction

T-cell lymphoproliferative neoplasms are heterogeneous, rare, and generally associated with poor outcomes. The most common peripheral T-cell lymphoma (PTCL) in North America, for example, is a diagnosis of exclusion, and remains “not otherwise specified (NOS)”. However, PTCL, NOS was recently demonstrated to include genetically and transcriptionally distinct subsets, one of which highly expresses the transcription factor GATA-3 [1, 2]. These findings have significant clinical implications, as GATA-3 expression was associated with chemotherapy resistance and dismal outcomes [3].

The GATA family of transcription factors regulate cell development and differentiation, and are so named, as they bind the DNA consensus motif WGATAR via two highly conserved zinc fingers, both of which have distinct roles in DNA binding and protein-protein interactions [4, 5]. GATA-1 and −2 are predominantly expressed by hematopoietic progenitor, erythroid, megakaryocyte, and various myeloid cells. Recurrent mutations in these GATA family members are associated with dyshematopoiesis and myeloproliferative disorders [6]. In contrast, GATA-3 expression is largely restricted to the lymphoid lineage among hematopoietic cells [7], where it regulates the differentiation and function of T-cell progenitors and mature T-cell subsets [8–16].

The observation that a PTCL, NOS subset highly expressed GATA-3, particularly in view of its long established role in Th2 cell differentiation [7], was ontologically interpreted, associating GATA-3 expression with a Th2 “cell of origin” in these lymphomas [1, 2]. Subsequent work demonstrated that “high-risk” chromosomal gains/losses, including p53 loss, are largely specific for, and highly prevalent among, GATA-3 expressing PTCL, and may explain (or contribute to) the poor outcomes observed in patients afflicted with these lymphomas [17]. Consequently, GATA-3 expression in these PTCL, beyond possibly informing disease ontology, has emerged as a potential biomarker for this genetically high-risk subset of PTCL, NOS [18]. However, in conventional (non-malignant) T cells, GATA-3’s role is not limited to T-cell differentiation, as GATA-3 also regulates cell growth and proliferation [8–16], thus suggesting that GATA-3 may have a previously uncharacterized oncogenic role in these lymphomas. If so, GATA-3 expression, and the transcriptional programs it directs, may have clinically and therapeutically significant implications that extend beyond PTCL, NOS. Therefore, we sought to determine whether GATA-3 is a bona fide proto-oncogene and to further characterize GATA-3 dependent transcriptional programs across the spectrum of immature and mature T-cell derived neoplasms.

## Materials and methods

### Patient specimens

Primary patient specimens were identified from institutional databases and those with archived formalin-fixed, paraffin-embedded (FFPE) tissue selected in accordance with US federal regulations and the Declaration of Helsink and with institutional review board (IRB) approvals. Primary malignant T cells for ex vivo studies were isolated from patients with either Sezary syndrome or PTCL with secondary leukemic involvement (Supplemental Table 2), as determined by concurrent clinical flow cytometric analysis.

### Cell lines

All cell lines were mycoplasma free and independently authenticated by short tandem repeat (STR) profiling, performed by ATCC (data not shown), and immunophenotyping (data not shown).

Additional details about methods and analyses are provided in the supplemental Materials.

## Results

### GATA-3 is highly expressed in selected T-cell neoplasms

**Fig. 1:**
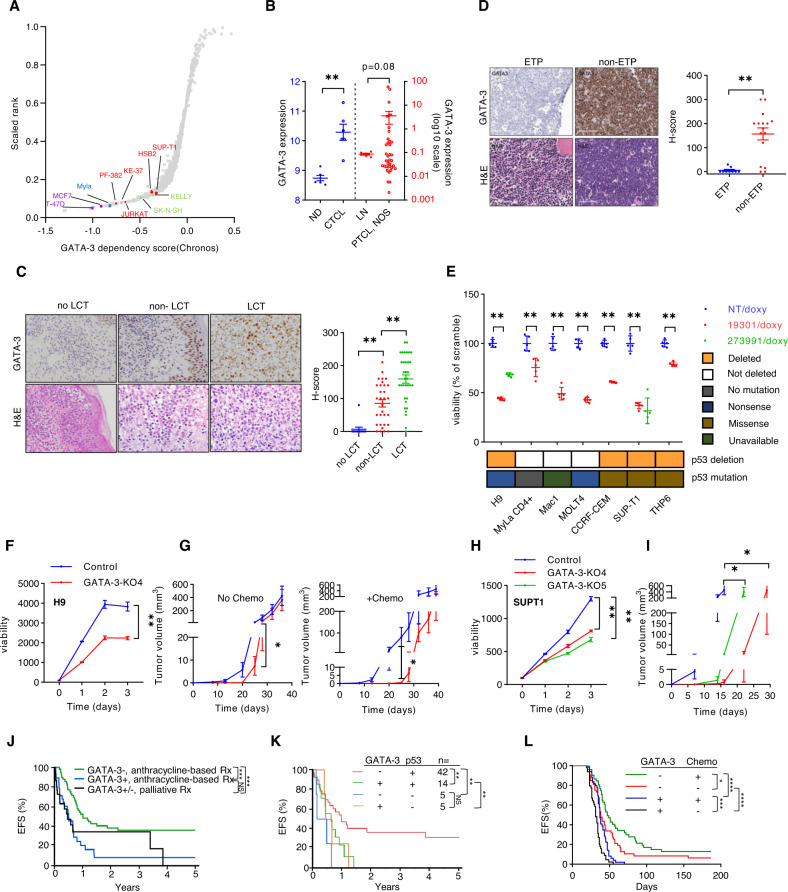
GATA-3 dependency in T-cell lymphoproliferative neoplasms. **A** A scatter plot showing relative dependency on GATA-3 in T-cell acute lymphoblastic leukemia (T-ALL) cell lines (*n* = 5, in red), a mature T-cell lymphoma cell line (*n* = 1, in blue), and 1048 additional cell lines, including breast cancer (in purple) and neuroblastoma (in green) cell lines, for which GATA-3 is a known dependency. The Y-axis shows the GATA-3 dependency rank and the X-axis shows the GATA-3 dependency score from Chronos (21Q3) for each individual cell line. **B** Expression of GATA-3 transcripts were collected from three microarray datasets. Two datasets from Normal donors (ND) and Sézary syndrome (CTCL) patient samples are shown at left (in Gene Expression Omnibus database, accession number: GSE131738 and GSE39041), and a dataset including reactive lymph nodes (LN) and PTCL, NOS biopsies is shown at right (accession number: GSE36172). **C** GATA-3 immunohistochemistry and hematoxylin and eosin (H&E) staining was performed in skin biopsies obtained from CTCL patients with limited-stage (patch/plaque) disease that never developed large cell transformation with clinical follow-up (no LCT, *n* = 12), and in paired biopsies obtained from patients before (non-LCT, *n* = 31) and after LCT (LCT, *n* = 34). Representative examples are shown (at left) and the data summarized (at right). **D** GATA-3 immunohistochemistry was similarly performed in a cohort of T-ALL biopsies, including early thymocyte progenitor (ETP, *n* = 9) and non-ETP specimens (*n* = 16). Representative images are shown (at left) and the data summarized (at right). **E** CTCL and T-ALL cell lines were transduced with doxycycline-inducible constructs expressing GATA-3 (19301, 273991) or non-targeting (NT) shRNA. Relative cell viability was determined 7 days after GATA-3 knockdown. As comparable results were achieved with two independent shRNA in H9 and SUPT-1 cells, the remaining cell lines were transduced with the GATA-3 targeting shRNA (19301, in red) associated with the most significant GATA-3 knockdown. P53 deletions and mutations are prevalent in these cell lines, and are indicated below. **F** CRISPR/cas9-mediated GATA-3 knockout (KO) was achieved in H9 cells (KO4), and cell proliferation and viability determined by RealTime-Glo. Y-axis demonstrates luminescence intensity. **G** NSG mice were injected subcutaneously with control and GATA-3 KO H9 cells, and mice treated with cyclophosphamide and vincristine (or vehicle control) on days 24 and 31 (*n* = 10). Tumor volumes, stratified by treatment, are shown. **H**, **I** Cell proliferation and viability was similarly determined in two independent GATA-3 KO SUP-T1 subclones (**H**) and tumor volumes (**I**) measured in NSG mice bearing control (*n* = 8) and GATA-3 KO xenografts (*n* = 6). Y-axis demonstrates luminescence intensity. **J**, **K** A cohort of PTCL, NOS patients was stratified by GATA-3 expression and treatments received, and event-free survival (EFS) examined (**J**). Loss of the p53 locus (17p13.1) was determined by FISH in the subset of cases for which tissue had not been exhausted, and EFS similarly examined (**K**). **l** Event-free survival (EFS) from Splenocytes from lymphoma-bearing GATA-3^fl/fl^ (*n* = 9) and GATA-3^+/+ or fl/+^ (*n* = 10) SNF5^fl/fl^, CD4-Cre mice were adoptively transferred to B6 recipients (*n* = 4–5/biologic replicate) and were treated with cyclophosphamide and vincristine (or vehicle control) every 7 days for 2–3 weeks, and EFS determined. Data are represented as mean ± s.e.m. (standard error of the mean). *p < 0.05, **p < 0.01, ***p < 0.001, ****p < 0.0001.

To initially investigate whether GATA-3 is a dependency in T-cell lymphoproliferative neoplasms, GATA-3 dependency scores were generated in DepMap. Neuroblastoma and breast cancer cell lines, for which a GATA-3 dependency has been established [19, 20], were identified. Comparable dependency scores were observed in six cell lines generated from patients with T-cell lymphoproliferative neoplasms derived from either immature (T-cell acute lymphoblastic leukemia; T-ALL) or mature (cutaneous T-cell lymphoma; CTCL) T cells (Fig. 1A). In comparison with conventional (non-malignant) T cells, GATA-3 transcripts were more abundant in malignant T cells (Fig. 1B, Fig. S1A). Therefore, we examined the prevalence of GATA-3 expression in both CTCL and T-ALL specimens. Most patients afflicted with CTCL have patch/plaque (or “limited”) stage disease and are managed with skin-directed therapies [21]. However, a minority of these patients will develop large cell transformation (LCT), a clinically aggressive and genetically complex variant recognized by distinct histopathologic findings and associated with inferior survival [22]. GATA-3 expression by immunohistochemistry (Fig. 1C) was examined in limited-stage CTCL patients without LCT (mean follow-up 8.0 years, IQR: 3.8–10.4 years), and minimal GATA-3 expression observed (Fig. 1C). In contrast, GATA-3 expression, while prevalent in paired biopsies from limited-stage patients both prior to and at the time of LCT, was significantly increased in LCT biopsies, potentially implicating GATA-3 in LCT.

We next turned our attention to T-ALL. Despite the well-established role of GATA-3 in thymic selection and the development of T-ALL in mice transgenically expressing GATA-3 in T-cell progenitors [23], its role in T-ALL is poorly understood. Therefore, GATA-3 expression was examined in T-ALL derived from early T-cell progenitors (ETP) and those derived from more mature thymic immigrants (non-ETP). GATA-3 expression was largely restricted to non-ETP T-ALL (Fig. 1D), consistent with the demonstration of DNA methylation and epigenetic silencing of GATA-3 in ETP T-ALL [24], and recurrent loss-of-function GATA-3 mutations observed in this subset [25].

### GATA-3 is a dependency in p53-deficient T-cell neoplasms

Given the GATA-3 dependency observed, its expression across the continuum of T-cell neoplasms derived from immature (thymic) and mature (post-thymic) T cells, and its association with a clinically aggressive variant in CTCL, we performed doxycycline-inducible shRNA-mediated knockdown studies in a panel of well characterized CTCL (*n* = 3) and T-ALL (*n* = 4) cell lines using previously characterized shRNA [(1), and Fig. S1B]. Given the association between GATA-3 expression and p53 deletions in PTCL, NOS [1, 2, 17], these cell lines were stratified by p53 status (Fig. S1C). A significant decrease in viability was observed upon GATA-3 knockdown regardless of p53 status (Fig. 1E). In order to exclude potential off-target effects, CRISPR-Cas9 GATA-3 knockout was performed in two representative cell lines (Fig. S1D). GATA-3 knockout in H9 cells was associated with decreased cell growth in vitro (Fig. 1F) and delayed engraftment and a transient reduction in xenograft growth in vivo (Fig. 1G). However, long-term culture (data not shown) and xenograft growth led to the competitive outgrowth of GATA-3 expressing clone(s) (Fig. S1E), consistent with selection pressure favoring GATA-3 expressing subclones. Therefore, GATA-3 knockout was similarly achieved in SUPT-1 cells, but subclones generated to ensure stable knockout. GATA-3 knockout was similarly associated with diminished cell growth in vitro (Fig. 1H) and in vivo (Fig. 1I, Fig. S1F). Furthermore, restoration of GATA-3 expression in these cells reversed this phenotype (Fig. S1G). In light of these findings, we examined the prognostic significance of GATA-3 expression in a large PTCL, NOS cohort (and patient characteristics are summarized in Supplemental Table 1). Consistent with prior studies [1, 2], GATA-3 expression was associated with a significant reduction in both event-free (Fig. 1J) and overall survival (Fig. S1H). A significant difference in survival between GATA-3 PTCL patients receiving the current standard of care in the frontline setting (i.e., an anthracycline-based regimen) and those receiving palliative therapies (most commonly hospice care, summarized in Supplemental Table 1) was not observed, highlighting the need for improved therapeutic strategies in patients with GATA-3 PTCL. Sufficient tissue was available for a subset of these patients, and the presence of p53 deletion was determined by FISH (Fig. S1I). The dismal outcomes associated with GATA-3 expression were similarly observed, regardless of p53 status (Fig. 1K). Collectively, these findings are consistent with previous studies suggesting that GATA-3 confers chemotherapy resistance in mature T-cell lymphomas [3]. To further address this possibility, we exploited a genetically engineered mouse model in which SMARCB1 (*SNF5*), a component of the SWI/SNF complex, is conditionally deleted in T cells using CD4-Cre [26], and crossed these mice with GATA-3 [27] and p53 floxed mice, thus establishing GATA-3^+^ (+/+, flox/+) and GATA-3^-^ (flox/flox) MTCL-bearing mice (Fig. S1J) in p53 proficient and deficient contexts. This model was selected as components of the SWI/SNF complex, including SMARCB1, are recurrently lost in mature T-cell lymphomas (MTCL) [17, 28, 29]. As previously reported [26], clonal MTCL, identifiable by the dominant expansion of T cells expressing specific T-cell receptor-Vβ chains, develop in these mice with complete penetrance and rapidly engraft in normal B6 recipients upon adoptive transfer [26]. Upon adoptive transfer, prolonged survival was observed in GATA-3 deficient lymphomas whether or not recipient mice were treated with cyclophosphamide and vincristine (Fig. 1L). While long-term survivors were rarely observed among GATA-3 deficient lymphomas, preserved GATA-3 expression was uniformly fatal, and chemotherapy administration associated with a clinically insignificant prolongation in survival. Furthermore, consistent with our findings in human PTCL, NOS (Fig. 1J), the prolonged survival observed in GATA-3 deficient lymphomas in this model was appreciated, albeit to varying degrees, irrespective of p53 status (Fig. S1K).

### GATA-3-dependent transcription promotes the growth and survival of neoplastic T cells

**Fig. 2:**
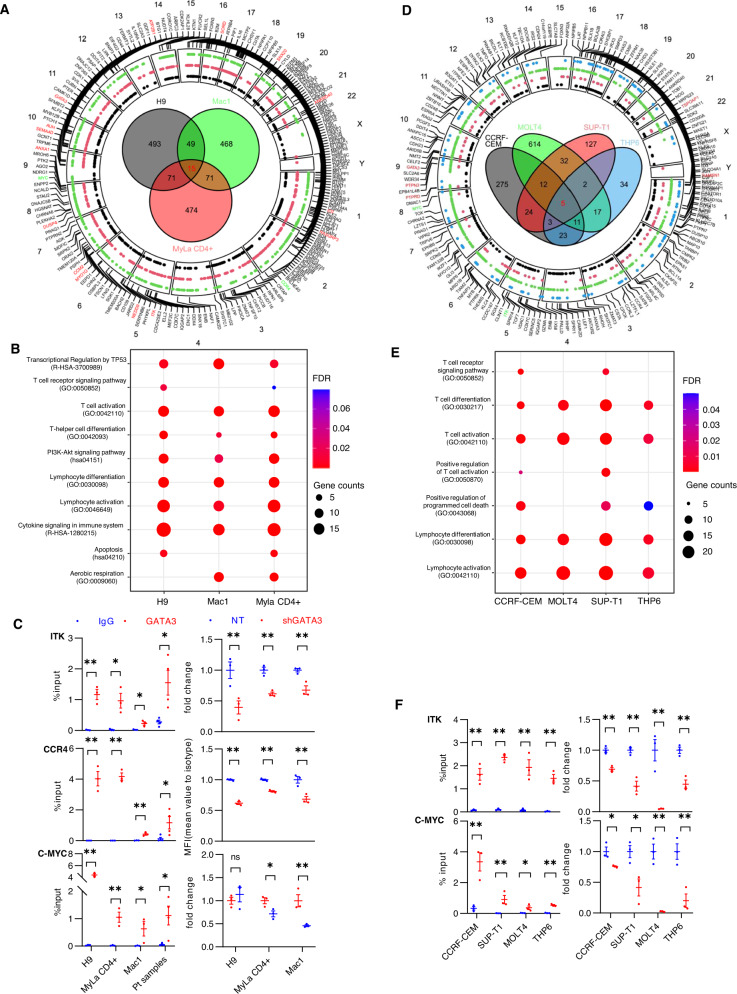
GATA-3 target genes identified in CTCL and T-ALL regulate cell growth and viability. **A** Circus plot showing GATA-3 target genes and chromosome location in CTCL cell lines. Target gene names identified in Mac1 (green), MyLa CD4 + (red), H9 (black), respectively, are indicated with the dots shown. Venn diagram of GATA-3 target genes in each cell line is shown, and target genes shared across all three cell lines are shown in red. Target genes used for validation are also shown in green. **B** Dot plots of representative pathways enriched with GATA-3 target genes in CTCL are indicated. **C** GATA-3 ChIP and binding enrichment at selected GATA-3 target genes (ITK, CCR4, and c-MYC) is shown (at left) for the 3 CTCL cell lines indicated, and for malignant T cells obtained from Sezary syndrome patients (*n* = 4). Gene expression upon GATA-3 knockdown (at right) was determined by qRT-PCR for ITK (top) and C-MYC (bottom). CCR4 expression was determined by flow cytometry and ∆MFI reported. Percent input (%input) was used as Y-axis in binding enrichment. Fold change was normalized to total GAPDH levels. **D** Circus plot showing GATA-3 target genes and chromosome location in T-ALL cell lines. Target gene names identified in THP6 (blue), SUPT1 (red), MOLT4 (green) CRF-CEM (black), respectively, are indicated with the dots shown. Venn diagram of GATA-3 target genes in each cell line is shown, and target genes shared across all four cell lines are show in red. Target genes used for validation are also shown in green. **E** Dot plots of representative pathways enriched with GATA-3 target genes in T-ALL are indicated. **F** GATA-3 ChIP and binding enrichment at selected GATA-3 target genes (ITK and c-MYC) is shown (at left) for the 4 T-ALL cell lines indicated. Gene expression upon GATA-3 knockdown (at right) was determined by qRT-PCR for ITK (top) and C-MYC (bottom). Percent input (%input) was used as Y-axis in binding enrichment. Fold change was normalized to total GAPDH levels. Data are represented as mean ± s.e.m (standard error of the mean). *p < 0.05, **p < 0.01.

As GATA-3-dependent transcription is context dependent [30], we performed an integrative analysis of GATA-3 ChIP-seq and RNA-seq datasets (Supplemental Tables 5, 6). A previously validated antibody for GATA-3 ChIP was utilized [31], and its binding specificity confirmed (Fig. S2A). Across CTCL and T-ALL cell lines, GATA-3 binding was largely restricted to intergenic and intronic sequences (Fig. S2B) harboring its canonical binding motif (Fig. S2C). Runx [32], STAT [33], and SMAD [34] binding motifs, all of which regulate gene transcription cooperatively with GATA-3, co-occurred with GATA-3 binding sites (Fig. S2C). Consistent with prior studies in conventional (non-malignant) T cells [30], GATA-3 binding peaks were distributed near epigenetic marks (H3K27Ac and H3K4Me1) associated with active or poised regulatory elements (Fig. S2D). An integrative analysis of the ChIP-seq and RNA-seq datasets was performed in CTCL cell lines (Fig. 2A) to identify GATA-3 target genes (Supplemental Table 7). The target genes identified were intimately involved in T-cell growth and survival (Fig. 2B), and consistent with prior studies, included TCR- and PI3K/AKT-mediated signaling pathways [2, 3]. We selected therapeutically relevant GATA-3 targets ITK [3], CCR4 [35], and c-Myc [2] for further validation. GATA-3 binding was enriched at these loci by targeted GATA-3 ChIP in both cell lines and primary T-cell lymphoma specimens (Fig. 2C). In addition, target gene expression was reduced upon GATA-3 knockdown (Fig. 2C). As prior studies have suggested that GATA-3 expressing mature T-cell lymphomas may be ontologically derived from Th2 cells [1], we examined the relationship between the GATA-3 target genes we identified and those associated with differentiated subsets of CD4 + T cells (Fig. S2E). Only a minority of the target genes identified were associated with Th2 specific genes. While this observation does not exclude an ontological relationship between Th2 cells and GATA-3 expressing MTCL, it certainly does highlight the context dependent nature of GATA-3 dependent transcription. A similar approach was adopted in representative T-ALL cell lines, whereby shared GATA-3 target genes were identified (Fig. 2D), including those involved in T-cell growth and survival (Fig. 2E), some of which (e.g., ITK, c-Myc) are therapeutically relevant and were subsequently validated (Fig. 2F).

**Fig. 3:**
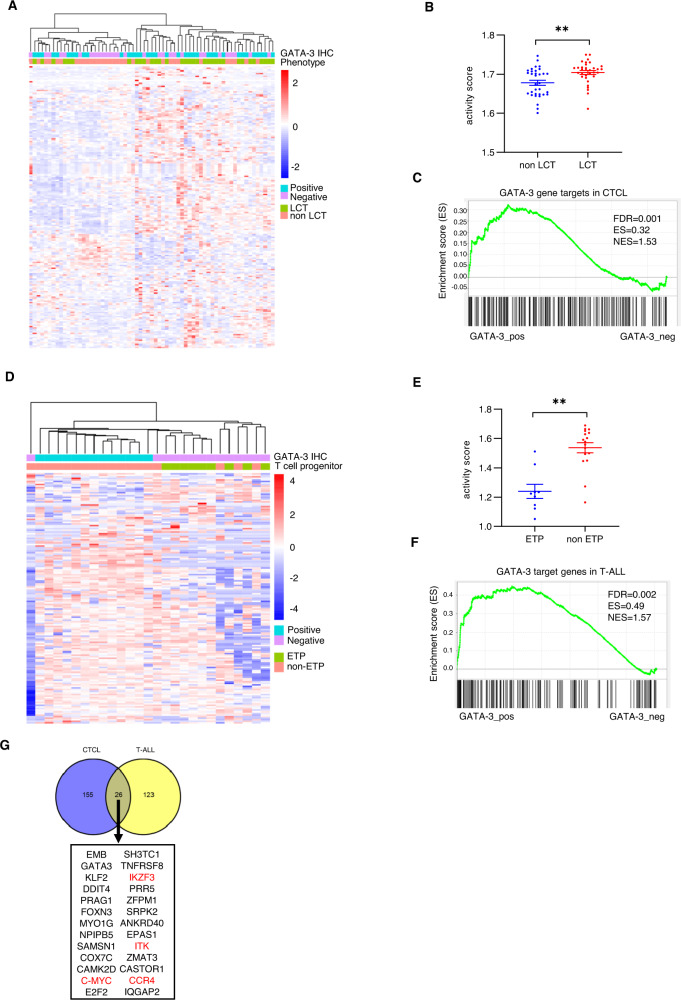
GATA-3 targets genes are highly regulated in CTCL and T-ALL cohorts. **A** Unsupervised hierarchical clustering was performed using GATA-3 target genes identified in CTCL cell lines in a cohort of CTCL biopsy specimens, including those with and without large cell transformation (LCT), as indicated. Immunohistochemistry for GATA-3 was performed, and cases stratified by GATA-3 expression, as indicated. **B** Scatter plot showing GATA-3 target gene activity score for non-LCT (*n* = 31) and LCT (*n* = 34) CTCL specimens is shown. **C** GSEA analysis of CTCL cohorts for GATA-3 target genes in CTCL. **D** Unsupervised hierarchical clustering was performed using GATA-3 target genes identified in T-ALL cell lines in a cohort of T-ALL biopsy specimens, including those with and without early thymocyte progenitor (ETP), as indicated. Immunohistochemistry for GATA-3 was performed, and cased stratified by GATA-3 expression, as indicated. **E** Scatter plot showing GATA-3 target gene activity score for non-ETP (*n* = 16) and ETP (*n* = 9) T-ALL specimens is shown. **F** GSEA analysis of T-ALL cohorts for GATA-3 target genes in T-ALL. **G** Venn diagram showing overlapping target genes between CTCL and T-ALL. Target genes identified in two out of three CTCL cell lines and in two out of four T-ALL cell lines were included for analysis. GATA-3 target genes of therapeutic interest are highlighted in red. Data are represented as mean ± s.e.m. (standard error of the mean). **p < 0.01.

In order to extend our findings to primary patient specimens, we performed RNA-seq in a CTCL cohort that included patients with and without LCT. Consistent with our prior findings, GATA-3 expression, as determined by immunohistochemistry, was associated with LCT (Fig. 3A). We restricted our analysis to the GATA-3 target genes we identified in cell lines and performed an unsupervised analysis in this cohort. Not surprisingly, LCT and non-LCT cases were transcriptionally distinct (Fig. 3A), and GATA-3 target genes were significantly enriched in LCT (Fig. 3B, C). We adopted a similar strategy in a cohort of T-ALL specimens. Non-ETP and GATA-3 expressing T-ALL specimens were transcriptionally distinct (Fig. 3D), and a significant enrichment in GATA-3 target genes was observed in non-ETP cases (Fig. 3E, F). Among the GATA-3 target genes identified, 26 were shared among immature (T-ALL) and mature (CTCL) T-cell neoplasms (Fig. 3G).

### IL-2 inducible T-cell kinase (ITK) is a therapeutic vulnerability in GATA-3-driven T-cell neoplasms

**Fig. 4:**
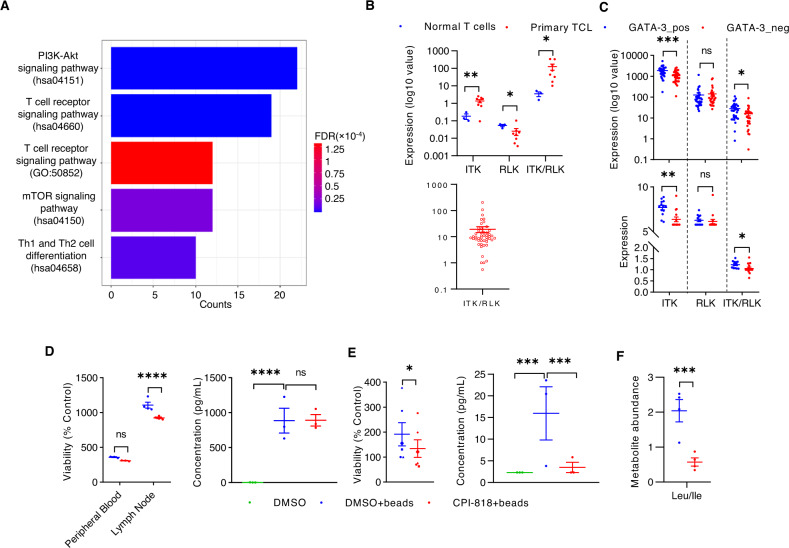
ITK is a therapeutic vulnerability. **A** Signaling pathways enriched with GATA-3 dependent kinases (*n* = 207) are shown. **B** ITK and RLK expression was determined in normal and malignant T cells (at top). The ITK/RLK ratio is also calculated and shown in an independent Nanostring dataset (at bottom). **C** ITK and RLK expression from CTCL (at top) and T-ALL (at bottom) biopsy specimens are summarized and stratified by GATA-3 expression as determined by immunohistochemistry. **D**, **E** Cell viability (left) and IL-10 production (right) are evaluated in normal T cells (**D**) and malignant T cells (**E**) isolated from peripheral blood. T cells were treated with anti-CD3/CD28 beads and CPI-818 (1 μM), or vehicle control (DMSO), as indicated. **F** Leucine/isoleucine abundance was quantified by mass spectrometry in similarly treated malignant T cells. Data are represented as mean ± s.e.m. (standard error of the mean). *p < 0.05, **p < 0.01, ***p < 0.001, ****p < 0.0001.

The GATA-3 target genes shared across the continuum of T-cell neoplasms are therapeutically relevant (Fig. 3G), including immunomodulatory drugs (IMiDs) that lead to IKZF3 degradation [36, 37], and the CCR4 specific monoclonal antibody mogamulizumab [38]. Given the potential therapeutic significance of GATA-3 dependent protein kinases, we examined protein tyrosine and serine/threonine kinases among GATA-3 target genes identified in CTCL. We identified 207 GATA-3 dependent kinases, including those associated with T-cell receptor (TCR) signaling (Fig. 4A), and among these, five were previously identified in a shRNA-based loss-of-function (“Achilles heel”) screen performed in a CTCL (MyLa CD4 +) cell line (Fig. S3A) [39]. However, we were most intrigued, for a variety of reasons, by IL-2 inducible T-cell kinase (ITK), a Tec family kinase that was not included in the prior screen for protein kinase dependencies. First, TCR signaling plays an important role in T-cell lymphomagenesis [40] and GATA-3 expression [3, 41], and ITK is a critically important mediator of TCR signaling in both conventional and malignant T cells [42]. Inhibition of Bruton’s tyrosine kinase (BTK), an ITK homolog required for antigen-receptor signaling in B cells, has transformed the therapeutic landscape in many B-cell lymphomas [43–45]. Pre-clinical studies suggest that ITK inhibition may be a viable therapeutic strategy in MTCL [3], and a clinical trial investigating a first-in-class, selective and irreversible ITK inhibitor is ongoing in these patients (ClinicalTrials.gov Identifier: NCT03952078). Therefore, we elected to further scrutinize ITK in GATA-3-expressing MTCL.

In contrast to B cells, where BTK is the dominant Tec family kinase required for B-cell receptor signaling, ITK, and its close homolog RLK, both contribute to TCR signaling in a partially redundant fashion [reviewed in [46]]. Therefore, ITK expression was examined, and in contrast to RLK, preferential ITK upregulation was observed in malignant T cells (Fig. 4B, Fig. S3B), particularly those expressing GATA-3 (Fig. 4C). Predictably, GATA-3 knockdown substantially decreased ITK expression (Fig. S3C). Therefore, we investigated CPI-818, a first-in-class and selective ITK inhibitor, and confirmed that this agent inhibits ITK autophosphorylation (at Tyr180) and ITK-dependent PLCγ phosphorylation (Fig. S3D), NF-kB (p65) nuclear translocation (Fig. S3E), and TCR-dependent GATA-3 upregulation (Fig. S3F). TCR-induced proliferation and IL-10 production in conventional T cells was minimally impaired by CPI-818 treatment (Fig. 4D), presumably due to preserved RLK expression and function. In contrast, inhibition of TCR-induced proliferation and IL-10 production in malignant T cells treated with CPI-818 was more significant (Fig. 4E). TCR activation culminates in significant metabolic reprogramming (Fig. S3G, H) [47], including increased leucine uptake, likely secondary to TCR-induced transporter (SLC7A5 or LAT1) upregulation (Fig. S 3I) [3], required to satisfy increasing biosynthetic demands. To examine the effects of CPI-818 on metabolism, primary T-cell lymphoma (Sezary) specimens were profiled by liquid chromatography-coupled tandem mass spectrometry-based metabolomics. Upon CD3/CD28 stimulation, CPI-818 treatment decreased both leucine/isoleucine levels (Fig. 4F) and decreased SLC7A5 (LAT1) expression (Fig. S3I). Collectively, these data suggest that selective ITK inhibition may preferentially target GATA-3 driven malignant T cells while minimally impacting the adaptive T-cell immune response.

### The histone acetyltransferase p300 binds and acetylates GATA-3

**Fig. 5:**
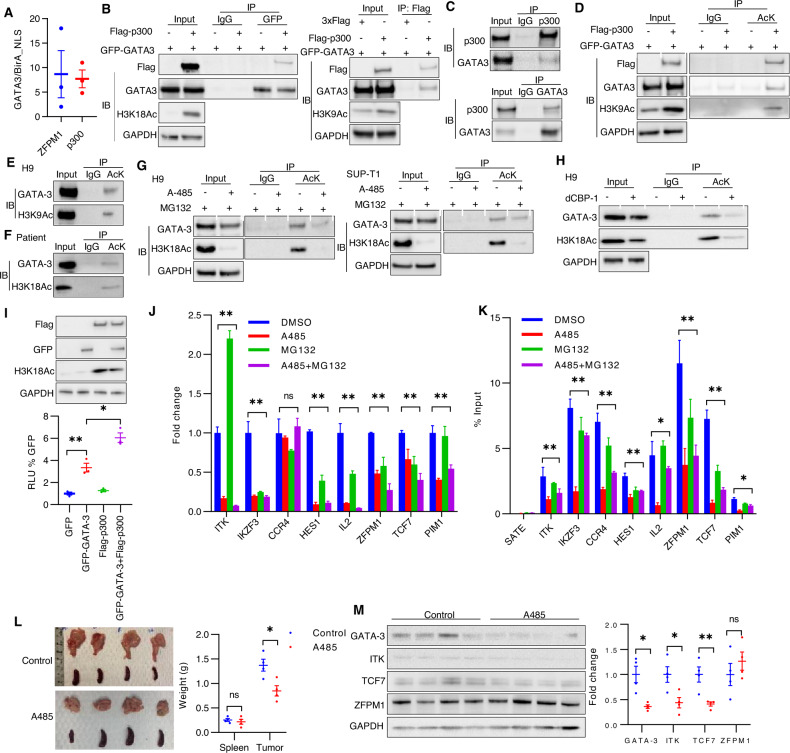
p300 interacts with and acetylates GATA-3. **A** Bio-ID identified P300 and ZFPM1 as GATA-3 binding partners. The ratio of biotinylated P300 and ZFPM1 peptides identified in GATA-3-BirA and control BirA-NLS transduced Jurkat cells is shown on the y-axis. **B** GFP-tagged GATA-3 and Flag-tagged P300 are expressed in HEK293T cells. Anti-GFP (or isotype control) immunoprecipitation (IP) is performed, and cell lysates immunoblotted (IB) for P300 (Flag) and GATA-3 (GFP), as indicated. H3K18 acetylation, as a surrogate for P300 acetyltransferase activity, was examined in the input, as indicated. **C** Endogenous GATA-3 and P300 are co-immunoprecipitated, as indicated, in H9 cells. **D** HEK293T cells transfected with GFP-tagged GATA-3 and Flag-tagged P300 are utilized and acetylated proteins immunoprecipitated, and GATA-3 identified in cell lysates by IB, as indicated. **E**, **F** Acetylation of endogenous GATA-3 is similarly examined by IP/IB in H9 cells (E) and malignant T cells obtained from a patient with Sezary syndrome (**F**). **G** GATA-3 acetylation is examined by IP/IB in H9 and SUP-T1 cells treated for 6 h with the acetyltransferase inhibitor A-485 and the proteosome inhibitor MG132, as indicated. **H** GATA-3 acetylation is similarly examined by IP/IB in H9 cells treated with dCBP-1, a P300 PROTAC. **I** Relative luciferase activity in HEK293T cells transfected with GFP-tagged GATA-3 and/or Flag-tagged P300, as indicated. Protein expression is examined by IB, and the corresponding blots are shown. **J**, **K** Analysis of representative GATA-3 target genes in H9 cells treated with A485 or/and MG132 by qRT-PCR (**J**) and GATA-3 DNA-binding by ChIP-qPCR (**K**), respectively. **L** The size of the spleen and tumor in PDX-bearing NSG mice randomly treated with A485 (100 mg/kg, i.p, consecutive 4 days) or vehicle control. Mice were euthanized on day 5 post-treatment. **M** Cell lysates are generated from individual PDX and GATA-3, ITK, TCF7 and ZFPM1 expression are examined by IB. Fold change is normalized to total GAPDH level and is summarized at right. Each symbol represents one mouse. Data are mean ± s.e.m. (standard error of the mean). ns, not significant, *p < 0.05, **p < 0.01.

GATA-3, like many transcription factors, lacks an obvious small molecule binding domain and is a challenging therapeutic target. In an effort to overcome this challenge, and as an alternative approach to targeting individual GATA-3 target genes, we sought to comprehensively characterize the GATA-3 interactome using an unbiased proximity-dependent biotin identification (BioID) approach. A mutated *E. coli* biotin ligase [BirA (R118G), hereafter referred to as BirA [48, 49], lacking substrate specificity was fused with GATA-3 using a doxycycline-inducible vector and expressed in Jurkat cells. BirA is predominantly cytoplasmic, thus a nuclear localization sequence (NLS) was added to ensure nuclear expression, and this BirA-NLS used as a negative control. Biotinylated proteins in BirA-GATA3 and BirA-NLS cells were enriched by affinity capture and identified by mass spectrometry. Using stringent selection criteria, 31 potential binding partners were identified, most of which were either transcriptional co-activators, repressors, or chromatin remodeling proteins, many of which are subject to recurrent mutations or copy number variants in T-cell neoplasms (Fig. S4A). The histone acetyltransferase p300 was identified (Fig. 5A), as was ZFPM1, a known GATA-3 binding partner [50]. Not surprisingly, intranuclear expression of p300 was universally observed by immunohistochemistry across a broad spectrum of T-cell neoplasms (data not shown). Flag-tagged p300 and GFP-tagged GATA-3 binding was demonstrated by co-immunoprecipitation (Fig. 5B), and the association of endogenous p300 and GATA-3 demonstrated in cell lines (Fig. 5C, Fig. S4B). As p300 is a large protein with multiple protein-protein interacting domains, including a bromodomain, truncated GATA-3 and p300 mutants were generated (Fig. S4C, E, G) in order to identify the p300 domain(s) required for GATA-3 binding. GATA-3 binding was localized to the c-terminal p300 domains, and did not require the p300 bromodomain (Fig. S4C-F), and was further localized to the IBiD domain (Fig. S4F). Furthermore, pharmacologic inhibition of the TAZ domain did not inhibit GATA-3 binding (Fig. S4I, J). GATA-3 binding to p300 was localized to the GATA-3 c-terminal, zinc-finger, and DNA-binding domains (Fig. S4G, H). These observations are notable, as inhibitors of p300 acetyltransferase and binding (e.g., bromodomain) domains are in development [51], and GATA family members may be post-translationally acetylated [52–56]. Therefore, we adopted multiple and complementary approaches to determine whether GATA-3 is a substrate for p300-mediated acetylation. A marked increase in GATA-3 acetylation was observed when overexpressed with p300 (Fig. 5D), and endogenous GATA-3 was acetylated in both cell lines (Fig. 5E, Fig. S5A) and a primary CTCL specimen (Fig. 5F). Next, we utilized A-485, a p300/CBP acetyltransferase inhibitor [57], but observed that A-485 led to a rapid reduction in GATA-3 protein (Fig. S5B), which we attributed to the well-described crosstalk between lysine acetylation and ubiquitination regulating protein stability [58]. Therefore, the proteasome inhibitor MG132 was included in these experiments, and prevented GATA-3 loss upon A-485 treatment (Fig. 5G, Fig. S5C), but without disrupting p300/GATA-3 binding (Fig. S5D). A significant reduction in GATA-3 acetylation was observed in cell lines treated with A-485/MG132 (Fig. 5G). We also utilized a recently described p300 PROTAC [i.e., dCBP-1 [59]] which degraded p300 within 5 h of treatment (Fig. S5F). A significant reduction in GATA-3 acetylation was similarly observed in cell lines treated with dCBP-1 (Fig. 5H, Fig. S5G). In order to overcome the challenge of target selectivity with these pharmacologic approaches, we were able to achieve, at best, partial p300 knockdown with a single shRNA, which led to diminished GATA-3 acetylation (Fig. S5E). Prior studies suggest that GATA-3-dependent transcriptional regulation may be functionally attenuated by GATA-3 acetylation [54, 56]. The demonstration that p300 and GATA-3 co-expression significantly increased expression of a GATA-3 dependent reporter when compared with GATA-3 expression alone supports this view (Fig. 5I). In order to more directly address this question, H9 cells were treated with A-485/MG132, as before, and GATA-3 dependent transcripts quantified. A significant reduction in 7 out of 8 transcripts was observed (Fig. 5J), and was associated with a significant reduction in GATA-3 binding at these loci, as determined by GATA-3 ChIP (Fig. 5K). Consistent with these findings, NSG mice bearing a PDX generated from a GATA-3^+^ PTCL, NOS patient, were treated with A-485 or vehicle control (Fig. 5L). A significant reduction in tumor size was appreciated (Fig. 5L), and loss of GATA-3 itself, and 2 out of 3 target genes examined, was observed (Fig. 5M). Collectively, these findings demonstrate that p300 binds and acetylates GATA-3, and further suggests that GATA-3 acetylation may be required for optimal transcriptional regulation of its target genes.

### GATA-3 acetylation regulates DNA binding

**Fig. 6:**
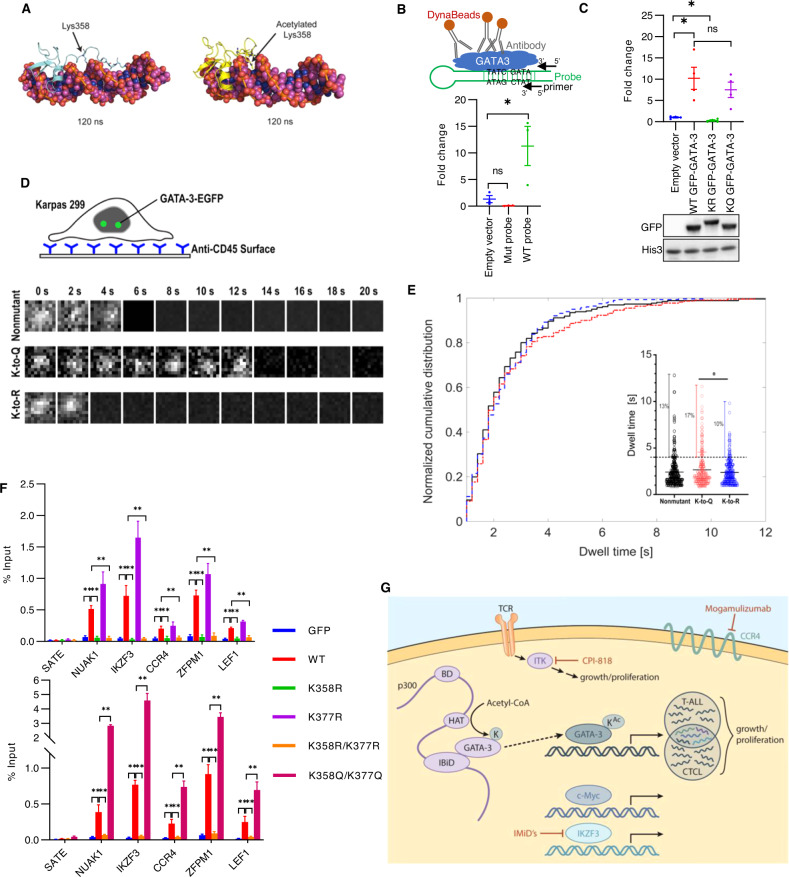
GATA-3 acetylation is required for DNA binding. **A** Molecular dynamics simulations are performed comparing GATA-3 that is unacetylated at K358 (left) or acetylated at K358 (right). **B** Schematic of the in silico GATA-3 DNA-binding assay is shown. GFP antibody recognizes GATA-3-DNA probe complex, which is immunoprecipitated using DynaBeads. Primers specific for the DNA probe are used to quantify the immunoprecipitated DNA probe in complex with GATA-3. GFP-tagged GATA-3 specifically binds to DNA probes containing a palindromic consensus GATA binding motif (WT probe), but not a DNA probe in which the GATA binding motif has been mutated (Mut probe). **C** Quantification of GATA DNA probe (WT probe) binding to wild type- (WT), K358R/K377R mutated- (KR), and K358Q/K377Q- mutated (KQ) GATA-3 expressed in HEK293T cells. A representative immunoblot from 4 independent experiments is shown. **D** Schematic of the immobilization of Karpas299 using anti-CD45 antibodies on a glass coverglass for stable single-molecule imaging. Representative time-lapsed single-molecule images of GFP-tagged WT, gain-of-function (GOF, K358Q/K377Q), and loss-of-function (LOF, K358R/K377R) GATA-3 are shown. **E** Distribution of GATA-3 dwell times (>1 s) measured by single-molecule microscopy in the wild type (black), GOF (K-to-Q, shown in red), and LOF mutant (K-to-R, shown in blue) Karpas299 cells. The insert shows the distribution of individual GATA-3 dwell times (s) for each cell line. Dashed line separates GATA-3 binding events that are at least 4 s long from shorter events. Fraction of events longer than 4 s: 13% for the wild type, 17% for the K-to-Q mutant, and 10% for the K-to-R mutant. **F** Analysis of GATA-3 binding to representative target genes in Karpas299 cells overpressing GFP alone, GFP-tagged WT-, K358R-, K377R-, K358R/K377R-, and K358Q/K377Q GATA-3 by GATA-3 ChIP-qPCR. **G** Model of GATA-3 acetylation and transcriptional regulation of therapeutically relevant target genes. Data are mean ± s.e.m. (standard error of the mean). ns not significant. *p < 0.05, **p < 0.01.

In order to further understand the putative role of GATA-3 acetylation in DNA binding, we performed mass spectrometry, and acetylation of two lysines (K358, K377), both located near the c-terminus of the c-terminal zinc-finger domain, were identified (Fig. S6A). Six lysines were not covered, hence acetylation of these lysines, or low-level acetylation of alternative lysine residues, cannot be excluded [54, 56, 60]. Nonetheless, molecular dynamic simulations suggest that acetylation at K358 reduces the interaction between this region and the DNA phosphate backbone, allowing for preferential positioning of GATA-3 within the DNA minor groove (Fig. 6A, Fig. S6B). Both loss-of-function (LOF, K358R/K377R) and gain-of-function (GOF, K358Q/K377Q) mutants were generated, and their ability to bind a consensus binding motif examined using a novel in silico assay. We constructed a stem-loop DNA probe containing a GATA consensus binding motif and sequences for PCR amplification (Fig. 6B). A mutated probe, in which the GATA binding motif was absent, was utilized as a negative control. The GATA-3 probe specifically bound GATA-3 in nuclear lysates (Fig. 6B). The K358R/K377R mutants were unable to bind this probe, whereas binding to the K358Q/K377Q mutants was preserved (Fig. 6C). Recognizing the significant limitations associated with an in silico approach, we subsequently performed single-molecule imaging in Karpas 299 cells, which do not express detectable endogenous GATA-3 (data not shown), expressing nonmutated, K358R/K377R, or K358Q/K377Q GATA-3 fused with EGFP (Fig. 6D). Sample images are shown (Fig. 6D), and GATA-3 dwell times presumed as DNA binding events were calculated (Fig. 6E). Dwell times less than one second were considered transient interactions and were excluded from analysis. We observed a slight increase in the mean GATA-3 dwell time of the gain-of-function (K358Q/K377Q; GOF) mutant compared to the loss-of-function (K358R/K377R; LOF) mutant and the nonmutant. Of note, there was a significant increase in the percent of GATA-3 binding events analyzed with dwell times greater than 4 s with the GOF mutant compared with the LOF mutant, and a non-significant increase compared to the nonmutant.

We next generated both single and double K-R and K-Q mutants and stably expressed these GFP-tagged mutants. We identified five GATA-3 gene targets in Karpas 299 cells that bound nonmutated GATA-3 by ChIP. In these cells, GATA-3 enrichment at these loci was abolished in the K358R mutant, thus demonstrating that acetylation at K358 is required for optimal DNA binding (Fig. 6F). In contrast, binding was not diminished, but increased, in the K377R mutant (Fig. 6F). In order to determine which acetylated site is dominant, we examined the corresponding double mutants. DNA binding was abolished with K358R/K377R, but significantly increased using the gain-of-function K358Q/K377Q mutant (Fig. 6F), suggesting that acetylation at these residues dynamically regulates the binding affinity of GATA-3 for its DNA targets. Collectively then, these findings demonstrate that p300-dependent acetylation of GATA-3 attenuates its DNA binding affinity and target gene expression.

## Discussion

While prior studies have demonstrated that GATA-3 is associated with genetically and clinically distinct subsets of T-cell lymphoproliferative neoplasms, the work presented here provides the first direct demonstration that GATA-3 is a bona fide proto-oncogene in these aggressive neoplasms. The demonstration that current treatment paradigms are largely futile, at least in GATA-3 expressing PTCL, further supports GATA-3’s utility as both a diagnostic and prognostic biomarker. However, and more importantly, the work presented here demonstrates that GATA-3’s utility should not be limited to these applications. Instead, GATA-3 itself, and a number of the target genes it transcriptionally regulates, a few of which we have highlighted herein, are rational therapeutic targets. Therefore, our findings not only identify GATA-3 as a relevant oncogenic driver in these malignances, but also have significant therapeutic implications.

Distinct enhancer landscapes among GATA-3-associated T-cell neoplasms likely explain the divergent GATA-3 target genes we identified across the continuum of immature and mature T-cell neoplasms. Despite this heterogeneity, the GATA-3 dependent transcriptional programs we identified converged on key cell growth and survival pathways (Figs. S7, S8), many of which have been previously implicated in both T-cell lymphomagenesis and chemotherapy resistance [61], including those that are T-cell receptor (TCR)- or PI3K/AKT-dependent [3, 40]. Consequently, GATA-3 expression was significantly associated with poor outcomes following the administration of conventional chemotherapeutic agents in PTCL, NOS and in a genetically engineered mouse (GEM) model. These latter findings demonstrate that current treatment paradigms, at least in the frontline setting, are suboptimal, if not futile, for these GATA-3 PTCL. Consistent with these findings, development of large cell transformation, a histopathologically distinct and chemorefractory form of CTCL, was associated with increased GATA-3 expression and target gene expression.

Next generation sequencing efforts, while demonstrating that GATA-3-associated T-cell neoplasms are genetically distinct, also demonstrate that “high-risk” mutations and copy number variants, including p53 loss, are preferentially and recurrently observed in these lymphomas. While the GATA-3 dependent transcriptional programs we identified would suggest that GATA-3 directly regulates the biology of these lymphomas, the genetic landscape of these lymphomas undoubtedly contributes to their aggressive natural history and poor responses to existing therapies. Therefore, p53 status was examined whenever possible in cell line and GEM models, and in our PTCL, NOS cohort. Collectively, our findings do not support the hypothesis that GATA-3 is merely a surrogate marker for a genetically high-risk group of lymphomas. Rather, GATA-3 transcriptionally reprograms these T-cell neoplasms, and by doing so, functions as a bona fide proto-oncogene. Furthermore, our observation that selected GATA-3 target genes, including c-Myc, CCR4, IKZF3 and ITK, are therapeutically targetable is noteworthy (Fig. 6G), as improved therapeutic strategies for these lymphomas are clearly needed.

Like other transcription factors, GATA-3 is a challenging therapeutic target, thus we sought to characterize its interactome by BioID. Our findings are generally consistent with the interactome previously identified in normal T cells [62], including the observation that transcriptional co-activators and corepressors, many of which are subject to recurrent gains and losses in T-cell neoplasms, respectively, were identified. However, we were most intrigued by p300, as post-translational acetylation of many GATA family members has been variously associated with regulating protein stability, protein-protein interactions, and DNA binding affinity [52, 63], and previous studies further suggest a regulatory role for post-translational acetylation in regulating GATA-3 function [56]. Consistent with the role of post-translational acetylation within the GATA family, we observed that p300 bound and acetylated GATA-3, and that GATA-3 acetylation at K358 was required for optimal DNA binding. While we cannot exclude the possibility of alternative acetylation sites beyond those we identified, our findings do demonstrate that GATA-3 is dynamically acetylated, and its acetylation state likely plays an important role in DNA binding. Therefore, the development of alternative therapeutic strategies targeting the GATA-3/p300 complex specifically, or GATA-3’s acetylation state generally, are justifiable in future studies.

In summary, GATA-3 induces transcriptional programs that promote cell growth and proliferation, and is thus a bona fide proto-oncogene in various T-cell neoplasms across the continuum of immature to mature T-cell leukemias/lymphomas. Selected GATA-3 gene targets, the p300/GATA-3 complex, and GATA-3 acetylation present novel therapeutic vulnerabilities. We hope the new insights described herein will prompt future development of targeted and more effective treatments for these T-cell neoplasms.

## Supplementary information

Supplemental material-Methods, figures and tables 1-4

Supplemental Table 5 List of GATA3-regulated genes in cell lines

Supplemental Table 6 List of genes bound by GATA3 in all cell lines

Supplemental Table 7 GATA3 target genes

Supplemental Video 1 nonacetylated

Supplemental Video 2 acetylated

## Data Availability

Sequencing data generated during the current study are available, as described in the Supplementary Methods. Additional datasets generated during the current study are available from the corresponding author on reasonable request.
