# Supplementary material for: GATA-3 is a proto-oncogene in T-cell lymphoproliferative neoplasms
Source: Blood Cancer J. 2022 Nov 4;12(11):149. doi: 10.1038/s41408-022-00745-y (PMC9633835; doi:10.1038/s41408-022-00745-y)
Supplement: Supplementary file 1 — Supplemental material-Methods, figures and tables 1-4 [file 41408_2022_745_MOESM1_ESM.pdf]

Supplementary Methods

Patients

Peripheral T-cell lymphoma, not other specified (PTCL, NOS), T-cell acute lymphoblastic leukemia (T-ALL) and cutaneous T-cell lymphoma (CTCL) cases were identified from institutional databases and those with archived formalin-fixed, paraffin-embedded (FFPE) tissue were selected. De-identified clinical data, including treatments received, response to treatment, and survival outcomes were available for the PTCL, NOS patients, and were provided from participating sites on standardized case report forms (Extended Data Table 1). Event-free survival was defined as the time from diagnosis to the time of disease progression, next treatment, or death, whichever occurred first. In the absence of an event, patients were censored at the time of last follow-up. Whenever possible, tissue microarrays (TMA) were constructed from FFPE tissue blocks with sufficient tissue and lymphoma content. TMA were not constructed from the CTCL cases utilized. For semi-quantitative analysis of GATA-3 expression by immunohistochemistry, the intensity of staining (no staining=0, dim=1, moderate=2, high=3) and percentage of cells staining (quantified in 10% increments) were multiplied to generate an H-score (range: 0-300). Cases with an h-score ≥100 were scored as GATA-3 positive. Primary malignant T cells for *ex vivo* studies were isolated from patients with either Sezary syndrome or PTCL, NOS with secondary leukemic involvement (Supplemental Data Table 2), as determined by concurrent clinical flow cytometric analysis. For *ex vivo* studies using primary T-cell lymphoma specimens, malignant T cells were sorted using CD3 or CD4 microbeads (Miltenyi), as previously described. Sorted cells were cultured with anti-CD3/CD28 Dynabeads (Thermo Fisher Scientific #11132D) at a 1:1 ratio, where indicated. These studies were conducted in accordance with US federal regulations and the Declaration of Helsinki, and with institutional review board (IRB) approvals. Cases were reviewed independently by at least two hematopathologists and classified in accordance with 2016 WHO criteria.

Cell lines

HEK293T (CRL-3216), H9 (HTB-176), SUP-T1 (CRL-1942), MOLT4 (CRL-1582), CCRF-CEM (CRM-CCL-119) cells were purchased from American Type Culture Collection (ATCC). MyLa CD4+ (95051032) were purchased from Millipore Sigma. Karpas 299 and Mac-1 cells were kindly provided by Dr. Megan Lim.THP6 cells were kindly provided Dr. Mark Chiang. T8ML-1 cells were kindly provided by Drs. Fujiwara and Yasukawa. HEK293T cells were cultured in DMEM supplemented with 10% FBS. Other cells were cultured in RPMI1640 supplemented with 10% FBS. CCRF-CEM and THP6 cells were maintained in the medium supplemented with 50 μM β-mercaptoethanol (Sigma-Aldrich #M3148). Inducible expression in each engineered cell line was performed by addition of doxycycline (10 ng/ml) to the growth medium. All cell lines were mycoplasma free and independently authenticated by short tandem repeat (STR) profiling, performed by ATCC (data not shown), and immunophenotyping (data not shown). The status of p53 in/del/SNV for each cell line was determined using both CCLE and COSMIC databases. CPI-818 was provided by Corvus Pharmaceuticals under a material transfer agreement. IL-10 ELISA was performed using cell-free supernatants. Cell viability was analyzed by RealTime-Glo^{TM} MT Cell Viability Assay (Promega #G9711) according to manufacturer's instructions. Briefly, 5,000 cells were mixed with substrate and NanoLuc^{TM} enzyme and seeded to 96-well plate in triplicate or quadruplicate. Luminescence was measure from day 0 to day 7. Data shown was performed in at least 3 independent experiments. For cell surface staining, cells were stained with fluorochrome-conjugated antibodies and washed before flow analysis. For intracellular staining, cells were fixed and permed using FOXP3 Fix/Perm Buffer Set (BioLegend

#421403) then stained with appropriate antibodies. Cells were washed before flow analysis. Flow cytometry data was acquired using CyAn™ ADP Analyzer (Beckman Coulter) and the data were analyzed by Summit 5.3 or FlowJo. Mean fluorescence value was used for protein expression in cells. All antibodies are listed in Supplemental Data Table 4.

Immunohistochemistry

Lymphoma tissue sections were deparaffinized and stained on BOND RX automated stainer (Leica Biosystems) using Bond Polymer Refine Detection kit (Leica #DS9800). Sections were subjected to EDTA-based antigen retrieval (BOND ER2 solution #AR9961) for 40 min at 100°C. Non-specific binding was blocked by incubating sections with Background Sniper protein block (Biocare Medical #BS966) for 15 minutes. Sections were then incubated with anti-GATA-3 rabbit monoclonal antibody (Cell Signaling #5852) at 1:100 dilutions for 30 minutes followed by signal detection with polymer-HRP-labeled secondary antibody and DAB. Sections were counterstained with hematoxylin for 10 min, dehydrated and mounted with Micromount media (Leica Microsystems #3801730). For semi-quantitative analysis, the intensity of staining (no staining=0, dim=1, moderate=2, high=3) and percentage of cells staining (quantified in 10% increments) were multiplied to generate an H-score (range: 0-300). Cases with an h-score ≥100 were scored as GATA-3 positive.

Mouse models and *in vivo* experiments

Mouse studies were approved by the University Committee on Care and Use of Animals (UCUCA) and performed in accordance with guidelines established by the Unit for Laboratory Animal Medicine (ULAM) at University of Michigan. Mice were housed under specific-pathogen free conditions. Treatment allocation was randomized, and all animals in given experiments were included for analysis. P53 floxed, SNF5 floxed, and CD4-Cre mice were obtained from Jackson Laboratory. GATA-3 floxed mice were kindly provided by Dr. Doug Engel.(1) SNF5 mice were provided on a mixed background, but were backcrossed for at least 10 generations onto a B6 background. All F1 mice were genotyped using tail DNA (Invitrogen Animal Tissue Direct PCR Kit). Mice were monitored two times weekly for event-free survival (EFS), where an event was defined as the development of easily palpable hepatosplenomegaly, bulky lymphadenopathy (>5mm), or any other condition requiring anesthesia. For adoptive transfer experiment, 5×10^6 bulk splenocytes obtained from lymphoma-bearing donor mice were retro-orbitally injected into 12-16 weeks old recipient C57BL/6 mice (Jackson Laboratory). We assumed that for recipients from a common donor, event times are normally distributed with a mean log(4) and standard deviation 0.3, thus assuring power at least 80% to detect a 60% relative change in EFS with the number of biologic and technical replicates included in the experiments performed. Any mice that developed periorbital tumors following adoptive transfer, likely due to technical inefficiency, and prior to study treatment, were excluded. Otherwise, mice were randomized (by cage assignment) to treatment, and investigators unblinded to treatment allocation. Where indicated, mice were treated with combined vincristine (0.5 mg/kg, i.p) and cyclophosphamide (40 mg/kg, i.p) weekly (two weeks after adoptive transfer) for 2-3 consecutive weeks, and mice followed for EFS. For the cell-line- and patient-derived xenografts studies, 2×10^6 (cell lines) or 5×10^6 (PDX) cells were injected subcutaneously on the shaved flanks of 12-16 weeks old female NSG mice (Jackson lab, strain #005557). The longest and shortest tumor dimension was measured once to twice weekly, and tumor volumes calculated using a modified ellipsoidal formula [volume = 0.5(length x width^2)]. For the PDX model, tumor-

bearing mice were randomized and treated with A-485 (100 mg/kg) or vehicle control intraperitoneally for four consecutive days, and then euthanized on day 5. The tumors and spleens were weighed, and cell lysates generated from tumors for immunoblotting.

Genotyping

Genomic DNA is isolated from the tails of mice using proteinase K (Qiagen, #19131) and cell lysis solutions as per the manufacturer's recommendations. After vortexing, tails are incubated overnight at 55°C in a bead bath. The next day, protein precipitation solution (Promega, #A1120) is added, samples vortexed, and then incubated on ice for at least 5 minutes. After centrifugation, DNA is precipitated from supernatants by 100% isopropanol, and then washed with 70% ethanol. After drying in a heating block, the DNA is suspended in DNase free water for PCR using Taq DNA polymerase, primers, and PCR master mix (Invitrogen, #18038042). The primer sequences utilized are shown in the Extended Data Table 3.

Cell lines

HEK293T (CRL-3216), H9 (HTB-176), SUP-T1 (CRL-1942), MOLT4 (CRL-1582), CCRF-CEM (CRM-CCL-119) cells were purchased from American Type Culture Collection (ATCC). MyLa CD4+ (95051032) were purchased from Millipore Sigma. Karpas 299 and Mac-1 cells were kindly provided by Dr. Megan Lim.THP6 cells were kindly provided Dr. Mark Chiang. T8ML-1 cells were kindly provided by Drs. Fujiwara and Yasukawa, and were cultured as previously described.(2) HEK293T cells were cultured in DMEM supplemented with 10% FBS. Other cells were cultured in RPMI1640 supplemented with 10% FBS, as previously described (3). CCRF-CEM and THP6 cells were maintained in the medium supplemented with 50 μM β-mercaptoethanol (Sigma-Aldrich #M3148). Inducible expression in each engineered cell line was performed by addition of doxycycline (10 ng/ml) to the growth medium. All cell lines were mycoplasma free and independently authenticated by short tandem repeat (STR) profiling, performed by ATCC (data not shown), and immunophenotyping (data not shown). The status of p53 in/del/SNV for each cell line was determined using both CCLE and COSMIC databases. Nutlin-3 was purchased from Selleckchem (S1061). Cells were treated with 5 μM nutlin-3 for 16 hours at 37 °C. A-485 and MG132 were purchased from Selleckchem (S8740 and S2619). Cells were treated with 2 μM A485 and/or 10 μM MG132 for 6 hours at 37 °C. Chetomin was purchased from APExBIO (A4505). dCBP-1 was purchased from MedChemExpress (HY-134582). The cells were treated with 1 μM Chetomin for 6 hours or 1 μM dCBP-1 for 5 hours at 37 °C. For cell selection, puromycin (Takara #631306) was utilized at concentrations (0.25-1 μg /ml) appropriate for each cell line. For *ex vivo* studies using primary T-cell lymphoma specimens (Extended Data Table 2), malignant T cells were sorted using CD3 or CD4 microbeads (Miltenyi), as previously described.(3, 4) Sorted cells were cultured with anti-CD3/CD28 Dynabeads (Thermo Fisher Scientific #11132D) at a 1:1 ratio, where indicated. CPI-818 was provided by Corvus Pharmaceuticals under a material transfer agreement. IL-10 sandwich ELISA was performed using cell-free supernatants (BioLegend).

GATA-3/p300 mutants and site-directed mutagenesis

Full-length or N-term (1-250 amino acids) or C-term (241-444 amino acids) GATA-3 was ligated into pLVX-AcGFP Vector (Clontech #632155), generating FL-, N-term, C-term GATA-3. Flag-tagged p300, N-term p300 and N-term △BD p300 were gifts from Dr. Sutapa Ray (Assistant Professor, University of Nebraska Medical Center). Flag-tagged C-term (1664-2414 amino acids) or C-term △ZZ (1709-2414 amino acids) or IBiD (1806-2414 amino acids) or TAZ2

(1709-1900 amino acids) p300 was ligated into pLVX-Puro Vector (Clontech #632164) separately. 3 x Flag-CMV vector (Sigma) was a gift from Luke Peterson (University of Michigan). All constructs were confirmed by Sanger sequencing. K358R, K377R, K358R/K377R and K358Q/K377Q mutants in FL-GATA-3 (pLVX-AcGFP GATA-3) were generated using a Quickchange XL Site-Directed mutagenesis Kit (Agilent Technologies #200517). Primers are provided in Extended Data Table 3. All mutants were confirmed by Sanger sequencing.

Transfection, lentiviral production and transduction

For Co-IP, 2.0 x10^6 HEK293T cells were cultured in a 10 cm-diameter dish overnight and cells were transient transfected with Flag-tagged p300 construct and GFP-tagged GATA-3 by PolyJet (SignaGen #SL100688) next day. The cell culture media was changed 16 hours later, and cell lysates were collected at day 2 post-transfection for the following experiment. For shRNA knockdown, shRNA plasmids were purchased from Sigma-Aldrich which express scrambled shRNA (SHC002), or shRNA that targets the p300 gene (TRCN0000231133, TRCN0000231134) and GATA-3 (TRCN0000019301, TRCN0000273991). Lentiviruses were produced from lentiviral vectors above. Briefly, 2500 µg of the above plasmid, 875 µg pMD2.G and 1625 µg psPAX2 were co-transfected into HEK293T cells. Lentiviruses were collected and precipitated according to the manufacture's instruction (TaKaRa #631232). 3 x 10^5 cells were infected with lentivirus in the presence of 8 µg/ml Polybrene (Santa Cruz #sc-134220). 3 days after infection, cells were selected in the presence of 0.25 µg /ml (Jurkat cells) or 1 µg /ml (H9, MyLa CD4+, Mac1, SUP-T1, CCRF-CEM and MOLT4) puromycin (TaKaRa #631306). After 10 days of puromycin selection, the cells were tested for p300 or GATA-3 expression by immunoblot. For overexpression of GATA-3 in Karpas 299 cells, pLVX-AcGFP, pLVX-AcGFP WT-, K358R-, K377R-, K358R/K377R-, K358Q/K377Q- GATA-3 constructs were packaged in HEK293T separately. 3 days after infection, cells were selected in the presence of 0.5 µg /ml puromycin. After 10 days of puromycin selection, the cells were tested for GATA-3 expression by immunoblot.

CRISPR/Cas9-based generation of GATA-3 depleted cell lines

For depletion of GATA-3 in human CTCL and T-ALL cell lines, 200,000 cells were transfected with 120 pmol guide RNA (IDT: Hs.Cas9.GATA3.1.AB, gRNA sequence: GGAGCTGTACTCGGGCACGT), 104 pmol Alt-R Cas9 enzyme and 100 pmol Alt-R Cas9 enhancer with a 4D-Nucleofector^{TM} system (Lonza) (parameter: EN-150 for H9 and CM-137 for SUP-T1). Cell clones were screened by FACS analysis (gate: ATTO^{TM} 550) and cultured in a limited dilution. Subclones were analyzed for GATA-3 expression by FACS analysis and immunoblot analysis.

BIO-ID Assay

GATA-3 cDNA was fused with biotin protein ligase harboring an R118G mutation (BirA) in the plVX-Tet-ON vector (Fig. S9) (5). A nuclear localization sequence (NLS) cDNA was similarly fused with biotin protein ligase (BirA-NLS). BirA-GATA-3 or BirA-NLS were ectopically expressed in Jurkat cell lines. BirA fusions were induced with doxycycline for 72 h, biotinylation was induced by adding 50 µM of Biotin for 24 h. Cell lysates were generated, and biotinylated proteins were precipitated utilizing streptavidin-sepharose beads (GE Healthcare #17-5113-01) before submission to MS/MS. GATA-3 binding partners were defined as those for which the mean ratio of BirA-GATA-3/BirA-NLS biotinylated peptides was >5 and the BirA-GATA-3/BirA-NLS ratio was >5 in at least 2 out of 3 technical replicates.

Dual-luciferase assay

The GATA-3 promoter (-148 bp to +587 bp), previously published,(6) was cloned into pGL4.10 vector (Promega). HEK293T cells were grown in 24-well plates and transfected with 0.2 µg pGL4.0-*GATA-3*-luc and 0.02 µg *Renilla* luciferase vector (pGL4.70; Promega), together with 0.2 µg pLVX-AcGFP (control) or 0.2 µg pLVX-AcGFP GATA-3 or/and 0.6 µg Flag-p300 plasmid as indicated. 48 hours after transfection, transfected HEK293T cells were harvested in Glo lysis buffer (Promega #E266A) and luciferase assays were performed using Dual-Luciferase reporter assay system (Promega #E2940). Firefly value was normalized to *Renilla* value. The relative luciferase activities (RLUs) were expressed as a percent of the pLVX-AcGFP GATA-3 or/and Flag-p300 plasmid transfection-induced luciferase activity vs. the pLVX-AcGFP plasmid transfection-induced luciferase activity. Three independent experiments were performed.

Immunoprecipitation (IP) and Immunoblot (IB)

Briefly, cells were lysed in 1 ml IP buffer (Thermo Fisher Scientific #87788) containing protease inhibitor (Thermo Fisher Scientific #78410) and 1 µl benzonase (Sigma-Aldrich #E1014) at 4°C for 2 h. After centrifuging at 13,000 r.p.m. for 10 min at 4°C, the supernatant was pre-cleared with ~16 µl Dynabeads Protein G (Invitrogen #10004D) for 1 hour at 4°C. Then, the protein concentration of the pre-cleared lysates was quantified by Pierce BCA protein assay (Thermo Fisher Scientific #23227). ~1.0 mg lysates were incubated with 2-4 µg of antibodies-conjugated Dynabeads at 4°C overnight. Following day, the lysates were washed 2 times with 0.9 ml PBS-T (0.05% Tween-20) at 4°C and boiled in elution buffer (0.1M Glycine, pH 2.8) and SDS gel sample buffer at 70°C for 10 min. For immunoblot, cells were lysed in RIPA buffer (Thermo Fisher Scientific #89900) containing protease inhibitor. After centrifuging at 13,000 r.p.m. for 10 min at 4°C, the protein concentration of the supernatant was quantified by Pierce BCA protein assay. 30~40 µg cell lysates were boiled in SDS gel sample buffer at 70°C for 10 min. Samples were then ran using 4%-12% NuPAGE Bis-Tris protein gels or 3%-8% Tris-Acetate protein gels (this gel was used for p300 detection) and transferred to PVDF membranes (Bio-Rad #1620177). Primary antibodies were used at 1:700 to 1:2000 dilutions for incubation overnight, and horseradish peroxidase (HRP) conjugated anti-mouse (Cell Signaling #7076s) or anti-rabbit (Cell Signaling #7074s) IgG antibodies were used at 1:1000. Blots were developed using SuperSignal West Femto (Thermo Fisher Scientific #34096). Primary antibodies used are provided in Extended Data Table 4.

GATA-3 DNA-binding assay

HEK293T cells were transfected with indicated vectors. Nuclear extract was prepared freshly from HEK293T cells according to manufacturer's instruction (Thermo Fisher Scientific #78835). The nuclear extract was pre-cleared with ~7 µl Dynabeads for 1 hour at 4°C and then quantified by Pierce BCA protein assay. Single stranded DNA oligos probe containing a palindromic GATA site (AGAATGTAGCCCTGGACTTCTCCCGCTCGCTATCAGATAAGGCCTTATUUCGATAAGGCCTTATCTGATAGCGAGCGGGAGAAGTCCAGGGCTACATTCT) or a mutant GATA-3 site (AGAATGTAGCCCTGGACTTCTCCCGCTCGCAAAAATTTTAGGCCTTATUUCGATAAGGCCTAAAATTTTTGCGAGCGGGAGAAGTCCAGGGCTACATTCT) were synthesized by Integrated DNA Technologies (Coralville, IA). For the binding reaction, 10 nM DNA oligo probe, 100 µg of nuclear extract were added to the PBS buffer containing proteinase inhibitor in a final assay volume of 200 µl. The binding assay was carried out at 4°C for 1 hour, and then 1 µg GFP antibody (Santa Cruz #sc-9996) was added for overnight. Following day, 8 µl Dynabeads was added to the assay mixture for 2 hours at 4°C. Non-specific bindings were washed twice with

200 µl PBS-T (0.05% Tween-20) at 4°C. GATA-3 protein and probe complex were eluted in 35 µl buffer (0.1M Glycine, pH 2.8) and heated at 70°C for 10 min. GATA-3 DNA binding abilities were measured using real-time PCR, which was performed on the BIO-RAD C1000 Thermal Cycler PCR instrument with 2× SYBR Green (Alkali Scientific Inc. #QS2020). Primer sequences used in this study were as follows: 5'-AGAATGTAGCCCTGGACTTC-3' and 5'-TTCTCCCGCTCGCTATCA-3'. Ct value was normalized to Ct value from the sample transfected with pLVX-AcGFP GATA-3 without probe. The relative GATA-3 DNA binding abilities were expressed as a fold change of pLVX-AcGFP GATA-3 vs. the pLVX-AcGFP. Three independent experiments were performed.

RNA isolation, sequencing and quantitative RT-PCR

RNAs were extracted using RNeasy Mini Kit (Qiagen #74106), and on column DNase I digestion (Qiagen, RNase-free DNase set #79254) was performed during extraction to avoid DNA contamination. RNA concentration was determined by NanoDrop™ One/Onec Spectrophotometer (Thermo Fisher Scientific). RNA was assessed for quality using the TapeStation (Agilent, Santa Clara, CA). Samples were prepared using the NEBNext Ultra II Directional RNA Library Prep Kit for Illumina (NEB #E7760L), Ribo depletion Module NEBNext rRNA Human/Mouse/Rat (NEB #E6310X) and NEBNext Multiplex Oligos for Illumina Unique dual (NEB #E6440L), where 100 ng of total RNA was ribosomal depleted using the rRNA Depletion module. The rRNA-depleted RNA is then fragmented 7 minutes determined by RIN (RNA Integrity Number) of input RNA as per protocol, and copied into first strand cDNA using reverse transcriptase and dUTP mix. Samples undergo end repair and dA-Tailing step followed by ligation of adapters. The products are purified and enriched by PCR to create the final cDNA library. Final libraries were checked for quality and quantity by TapeStation (Agilent) and Qubit (Thermo Fisher Scientific). This pool was subjected to 151bp paired-end sequencing according to the manufacturer's protocol (Illumina NovaSeq). Bcl2fastq2 Conversion Software (Illumina) was used to generate de-multiplexed Fastq files. For quantitative RT-PCR, complementary DNA (cDNA) was prepared using the QuantiTect Reverse Transcription Kit (Qiagen #205311). qPCR was performed using the Radiant SYBR Green H-ROX qPCR Kits (Alkali Scientific Inc. #QS2020) and analyzed using the ΔΔCt method with control samples set as 1. Primers used in this study were in the Extended Data Table 3.

Nanostring and immune gene expression profiling

Nanostring technology was performed as previously described. Briefly, RNA were extracted using RNeasy FFPE kit (Qiagen #73504) and measured using a Nanodrop 1000 instrument (ThermoFisher), 150 ng total RNA from each patient was analyzed using the Nanostring platform and the nCounter PanCancer Immune Profiling Panel (NS_CANCERIMMUNE_C2929) with downstream normalization and gene quantification performed using nSolver (v4.0).

Chromatin immunoprecipitation (ChIP)

ChIP was performed using SimpleChIP® Plus Enzymatic Chromatin IP Kit (Cell Signaling #9004) with minor modifications. Briefly, 5-10 million cells per IP were fixed using formaldehyde (1% final concentration) for 10 min at room temperature and were stopped with 125 mM glycine addition for 5 min at room temperature. After washing, cells were lysed in prepared Buffer A for 10 min at room temperature. After lysing, nuclei were collected (500 × g, 4°C, 3 min) and digested with prepared Buffer B and Micrococcal Nuclease for 20 min at 37 °C. After adding 50mM EDTA to stop digestion, nuclei were collected (16,000× g, 4°C, 1 min) and resuspend in

prepared ChIP buffer. Nuclei were then sonicated with two pulses of 40 seconds each and 30 s incubation on wet ice at setting 4 using an ultrasonic cell disruptor (Microson) with a 18-inch probe. Chromatin was clarified (16,000× g, 4°C, 10 min) and incubated with GATA-3 and IgG antibody overnight. Chromatin was then incubated with 30 µL Dynabeads Protein G (Invitrogen #10004D) for 4 hours and washed with low-salt washing buffer three times and high-salt washing buffer once. Elution and de-crosslinking were performed overnight in 150 elution buffer (300 mM NaCl, 5 mM DTT and 0.1% SDS in TE buffer, pH8.0) at 65 °C, and RNA and proteins were digested by adding RNase A and proteinase K, respectively. Eluted samples were purified by QIAquick^{TM} PCR Purification Kit (Qiagen #28104) and were ready for high-throughput sequencing. For ChIP-seq library preparation, NEBNext ChIP-Seq Library Prep Master Mix Set for Illumina (NEB #E6240) was used according to the manual's instructions. Library quality was determined by TapeStation (Aglient) prior to being sequenced on the Illumina NovaSeq-6000 platform. For ChIP-qPCR, same amount of purified DNA was tested using the Radiant SYBR Green H-ROX qPCR Kits (Alkali Scientific Inc. #QS2020) and analyzed using the ΔΔCt method with control samples set as 1. Primers used in this study are in the Extended Data Table 3. Three independent experiments were performed.

Cell viability

Cell viability was analyzed by RealTime-Glo^{TM} MT Cell Viability Assay (Promega #G9711) according to manufacturer's instructions. Briefly, 5,000 cells were mixed with substrate and NanoLuc^{TM} enzyme and seeded to 96-well plate in triplicate or quadruplicate. Luminescence was measure from day 0 to day 7. Data shown was performed in at least 3 independent experiments.

Flow Cytometry

For cell surface staining, cells were stained with fluorochrome-conjugated antibodies and washed before flow analysis. For intracellular staining, cells were fixed and permed using FOXP3 Fix/Perm Buffer Set (BioLegend #421403) then stained with appropriate antibodies. Cells were washed before flow analysis. Flow cytometry data was acquired using CyAn™ ADP Analyzer (Beckman Coulter) and the data were analyzed by Summit 5.3 or FlowJo. Mean fluorescence value was used for protein expression in cells. All antibodies are listed in Extended Data Table 4.

Fluorescence in-situ hybridization (FISH)

Evaluation for TP53 deletions was performed on formalin-fixed, paraffin-embedded sections using FISH with a probe set that includes a TP53 locus-specific identifier (17p13.1) and a chromosome 17 chromosome enumeration probe (Abbott, Abbott Park, IL). All cases were evaluated for adequacy by a pathologist board-certified in hematopathology (N.B.). 100 cells from each case were required for evaluation with a ratio of TP53:CEP17 < 0.8 required for evidence of deletion.

Protein Identification by LC-Tandem MS

In-gel digestion:

HEK293T cells were transfected with GFP-tagged GATA-3. 2 days post-transfection, cells were collected for IP with GFP antibodies. The samples were separated by SDS-PAGE and stained with SimplyBlue SafeStain (Thermo Fisher Scientific #LC6060). The protein samples were processed and analyzed at the Mass Spectrometry Facility of the Department of Pathology at

the University of Michigan. Gel slice corresponding to 76 MW (GFP was 26 MW and GATA-3 was 49 MW) was destained with 30% methanol for 4 h. Upon reduction (10 mM DTT) and alklylation (65 mM 2-Chloroacetamide) of the cysteines, proteins were digested overnight with 500 ng of sequencing grade, modified trypsin (Promega) at 37°C. Peptides were extracted by incubating the gel with 150 µl of 50% acetonitrile/0.1% TFA for 30 min at room temperature. A second extraction with 150 µl of 100% acetonitrile/0.1% TFA was also performed. Both extracts were combined and dried in a vacufuge (Eppendorf).

MS:

Resulting peptides were dissolved in 9 µl (for in-gel digest) and 2 µls of the peptide solution were resolved on a nano-capillary reverse phase column (Acclaim PepMap C18, 2 micron, 50 cm, Thermo Fisher Scientific) using a 0.1% formic acid/2% acetonitrile (Buffer A) and 0.1% formic acid/95% acetonitrile (Buffer B) gradient at 300 nl/min over a period of 90 min (2-25% buffer B in 45 min, 25-40% in 5 min, 40-90% in 5 min followed by holding at 90% buffer B for 5 min and re-equilibration with Buffer A for 30 min). Eluent was directly introduced into Orbitrap Fusion tribrid mass spectrometer (Thermo Fisher Scientific, San Jose CA) using an EasySpray source. MS1 scans were acquired at 120K resolution (AGC target=$1x10^6$; max IT=50 ms). Data-dependent collision induced dissociation MS/MS spectra were acquired using Top speed method (3 seconds) following each MS1 scan (NCE ~32%; AGC target $1x10^5$; max IT 45 ms).

Database Search:

Proteins were identified by searching the data against *Homo sapiens protein database* (20353 entries; reviewed; downloaded on 06/02/2019) using Proteome Discoverer (v2.3, Thermo Scientific). Search parameters included MS1 mass tolerance of 10 ppm and fragment tolerance of 0.2 Da; two missed cleavages were allowed; carbamidimethylation of cysteine was considered as fixed modification. Oxidation of methionine, deamidation of asparagine and glutamine, acetylation on lysine, arginine, serine, threonine, and tyrosine were considered as potential modifications. FixedPSM validator of the Proteome Discoverer was used to retain only higher quality PSMs ($\Delta Cn \geq 0.05$). All PSMs corresponding to the modified peptides were verified manually.

Snapshot metabolomics

Metabolites were extracted from cells by adding cold 80% methanol, incubating at -80°C for 10 minutes, followed by centrifugation at 10,000xg for 10 minutes at 4°C. The resulting metabolite supernatant were collected. Metabolite extracts were normalized to cell number, and the normalized fraction was transferred to a fresh 1.5mL tube and lyophilized by speedvac. Dried metabolite pellets were resuspended in a 50:50 mixture of MeOH and water.

Liquid chromatography-based targeted tandem mass spectrometry (LC-MS/MS)-based metabolomics were performed and the data analyzed as previously described.(7, 8) In brief, samples were run on an Agilent 1290 Infinity II LC -6470 Triple Quadrupole (QqQ) tandem mass spectrometer system consisting of the 1290 Infinity II LC Flexible Pump (Quaternary Pump), the 1290 Infinity II Multisampler, the 1290 Infinity II Multicolumn Thermostat with 6 port valve and the 6470 triple quad mass spectrometer. Agilent Masshunter Workstation Software LC/MS Data Acquisition for 6400 Series Triple Quadrupole MS with Version B.08.02 was used for compound optimization, calibration, and data acquisition.

Molecular dynamics simulations

X-ray crystal structure atomic coordinates of the C-terminal zinc finger of mouse GATA-3 bound to DNA were downloaded from the Protein Data Bank (PDB ID: 3DFV). A model of GATA-3 bound in the extended c-terminal conformation was created by taking the coordinates of DNA and protein chain D, with coordinates for residues 355 to 366 taken from conformation B. A model with acetylated Lys358 was also constructed by manually building the acetyl group using the Build tool in Maestro (Schrodinger Release 2020-3). The models were processed using Protein Preparation Wizard (Schrodinger Release 2020-3). Hydrogen atoms were added, bond orders were assigned, zero-order bonds to metals were assigned, and protein termini were capped with amide and acetyl groups. Protonation states were determined using PROPKA, the hydrogen bonding network was optimized, and the models were energy minimized with heavy atoms constrained to within 0.3 Å of initial position. Each model was placed in a volume-minimized orthorhombic TIP3P water box with distance buffers of 10 Å • 10 Å • 10 Å with periodic boundaries. In each case, the system was neutralized with K+ ions and salt concentration was set to 0.15 M KCl, and the OPLS3e force field was assigned. 500 ns of molecular dynamics simulation were carried out using DesmondGPU (Schrodinger Release 2020-3) for the Lys358 acetylated and non-acetylated models after treatment with the default equilibration protocol. Production phase of each simulation was conducted at 300 K in an NPT ensemble at a pressure of 1.01325 bar using the Langevin barostat with a 1.0 ps relaxation time and the Nose–Hoover thermostat with a 2 ps relaxation time with coupling style set to isotropic. The RESPA integrator was used with the time step set to 2 fs with initial randomized velocities set using the default seed (2007). Coulombic interactions were calculated using the useries method and the short-range cut-off was set to 9.0 Å. The recording interval of the trajectory was set to 100 ps.

Immobilization, preparation, imaging, and dwell time determination of EGFP-tagged GATA-3 in Karpas299

To make an immobilizing surface, wells of an 8-well Chamber Slide (Lab-TEK, Borosilicate sterile, No. 1.5, 155409) were coated with 150 µL of 1µg/mL anti-CD-45 antibody (Southern Biotech, 9625-01)) and incubated overnight at 4°C.
The coating solution was aspirated, then 50,000 Karpas cells were added to each well and incubated for 30 minutes in pre-warmed imaging buffer, containing 1% bovine serum albumin, 0.5 mM Ca^{2+}, 2 mM Mg^{2+}, and HBSS at 37 °C and 5% CO_2.

GATA-3 were imaged continuously with a 488nm laser in TIRF on a Nikon Eclipse Ti2-E inverted microscope (TIRF 100x, 1.49 NA objective lens) with 1.5x external magnification. The images were collected by a Photometrics Prime 95B sCMOS camera. GATA-3 images of fully immobilized Karpas 299 cells were acquired for 500-1000 frames at a power density of 133 Wcm^{-2} laser power and 200 ms exposure.

GATA-3-EGFP molecules that immobilized for at least 1 second (5 frames) were used for analysis. Dwell times were determined by calculating the number of frames individual GATA-3 stayed in frame and immobilized in the selected ROI, followed by calculating the time length in seconds. An unpaired t-test was used to determine significance.

Gene expression analysis

Raw reads were quality checked with FastQC (v0.11.8, http://www.bioinformatics.babraham.ac.uk/projects/fastqc/) and adapter trimmed with Cutadapt (v2.3).(9) Trimmed reads were mapped to the reference genome GRCh38 (ENSEMBL) using STAR (v2.7.8a) and assigned count estimates to genes using RSEM (v1.3.3) with default parameters.(10, 11) Differentially expressed genes were determined using expected counts by DESeq2 package (v1.32), p-value <-0.05 and fold-change >=1.5 or <=0.67 were considered statistically significant.(12) Heatmap were generated by pheatmap (v1.0.12) using 'average' clustering method. Pathway enrichment analysis was performed by Metascape (v3.0) with default parameters.(13) Activity Scores were calculated by Gene Set Variation Analysis (GSVA, v1.42.0) and in-house awk scripts. GATA-3 target gene set enrichment was analyzed by GSEA (v4.1.0), using Kolmogorov-Smirnov test and in-house gene targets database.(14, 15) Pathways were considered significant if FDR <= 0.25. Public datasets (GEO Accession number: GSE131738, GSE39041 and GSE36172) were also collected for GATA-3 expression plotting.

ChIP-sequencing analysis

Raw reads were mapped to reference genome GRCh38 (ENSEMBL) using bwa (v0.7.15) and all uniquely matched reads were retained.(16) Mapped reads were blacklist, PCR duplicates and multimapping reads filtered using samtools (v0.1.19).(16) Peak calling was performed by MACS2 (v2.1.2) to generate bed files with following parameters: --nomodel --extsize 50 -f BAMPE --keep-dup auto --min-length 100 -g hs -q 0.05 -B.(17) Called peaks were annotated by GREAT (v4.0.4) with default parameters.(18) Genome-wide distribution of binding peaks were determined in the reference to the gene list from genome GRCh38 by Homer (v4.1.0).(19) The loci were assigned to regions in TTS (transcription termination site), 5'UTR, 3'UTR, promoter, intron, exon and intergenic. Motif enrichment analysis was performed by Homer (v4.1.0) with default parameters. Heatmaps and profile plot for various ChIP-seq were created by Homer (v4.1.0) and Deeptools (v2.0).(20) Bed files from GATA-3 ChIP-seq and bigwig file from H3K4me1 and H3K27Ac ChIP-seq were used for building matrix and generating heatmap and profile plots. Each row plots the +/- 10kb region flanking the center of GATA-3 binding peaks. Circus plot and chordgram was generated by Circlize (v0.4.13), with T helper cell signature obtained from published paper.(21, 22) Other plots were generated in-house R scripts.

Integrated analysis

Overlap analysis between differentially expressed genes identified by DESeq2 and annotated genes identified by GREAT was firstly made. Then bed files were analyzed with whole gene expression by BETA (v1.0.7) to predict direct target genes and GATA's activating or repressive function.(23)

For gene dependency analysis, GATA-3 dependency score from Depmap website (https://depmap.org/portal/) were collected and their rank in individual cell line were calculated using in-house shell scripts. Dot plots and bar plots were generated by ggplot2 (v3.3.5).

Statistical analysis

GraphPad Prism 8.0 (GraphPad Software, San Diego, CA) and SPSS 13.0 (IBM, www.ibm.com/products/spss-statistics) software were used for data analysis. Clinical datasets were analyzed using JMP software (JMP, Cary, NC). Animal experiments were analyzed by independent t-tests (two-tailed) for tumor volume means, and by log-rank test for survival. p values less than 0.05 were considered statistically significant where, ns, not significant; $*p < 0.05$; $**p < 0.01$; $***p < 0.001$ and $****p < 0.0001$.

Data Availability

All raw and process ChIP-seq and RNA-seq data were deposited (GSE201744). Public available microarray datasets (GSE131738, GSE39041 and GSE36172) were analyzed as well.

REFERENCES

1. Hosoya T, Kuroha T, Moriguchi T, Cummings D, Maillard I, Lim KC, et al. GATA-3 is required for early T lineage progenitor development. J Exp Med. 2009;206(13):2987-3000.
2. An J, Fujiwara H, Suemori K, Niiya T, Azuma T, Tanimoto K, et al. Activation of T-cell receptor signaling in peripheral T-cell lymphoma cells plays an important role in the development of lymphoma-associated hemophagocytosis. Int J Hematol. 2011;93(2):176-85.
3. Wang T, Polk A, Lu Y, Wilcox RA. T-cell Receptor Engagement Confers Resistance to Chemotherapy in T-cell Lymphoproliferative Disorders. Blood (ASH Annual Meeting Abstracts). 2014;124(21):Abstract #2959.
4. Wang T, Lu Y, Polk A, Chowdhury P, Zamalloa CM, Fujiwara H, et al. T-cell Receptor Signaling Activates an ITK/NF-kappaB/GATA-3 axis in T-cell Lymphomas Facilitating Resistance to Chemotherapy. Clin Cancer Res. 2017;23(10):2506-15.
5. Lambert JP, Tucholska M, Go C, Knight JD, Gingras AC. Proximity biotinylation and affinity purification are complementary approaches for the interactome mapping of chromatin-associated protein complexes. J Proteomics. 2015;118:81-94.
6. Nakata Y, Brignier AC, Jin S, Shen Y, Rudnick SI, Sugita M, et al. c-Myb, Menin, GATA-3, and MLL form a dynamic transcription complex that plays a pivotal role in human T helper type 2 cell development. Blood. 2010;116(8):1280-90.
7. Halbrook CJ, Pontious C, Kovalenko I, Lapienyte L, Dreyer S, Lee HJ, et al. Macrophage-Released Pyrimidines Inhibit Gemcitabine Therapy in Pancreatic Cancer. Cell Metab. 2019;29(6):1390-9 e6.
8. Lee HJ, Kremer DM, Sajjakulnukit P, Zhang L, Lyssiotis CA. A large-scale analysis of targeted metabolomics data from heterogeneous biological samples provides insights into metabolite dynamics. Metabolomics. 2019;15(7):103.
9. Martin M. Cutadapt removes adapter sequences from high-throughput sequencing reads. . EMBnet journal. 2011;17(1):10-2.
10. Dobin A, Davis CA, Schlesinger F, Drenkow J, Zaleski C, Jha S, et al. STAR: ultrafast universal RNA-seq aligner. Bioinformatics. 2013;29(1):15-21.
11. Li B, Dewey CN. RSEM: accurate transcript quantification from RNA-Seq data with or without a reference genome. BMC bioinformatics. 2011;12:323.
12. Love MI, Huber W, Anders S. Moderated estimation of fold change and dispersion for RNA-seq data with DESeq2. Genome biology. 2014;15(12):550.
13. Zhou Y, Zhou B, Pache L, Chang M, Khodabakhshi AH, Tanaseichuk O, et al. Metascape provides a biologist-oriented resource for the analysis of systems-level datasets. Nature communications. 2019;10(1):1523.
14. Hanzelmann S, Castelo R, Guinney J. GSVA: gene set variation analysis for microarray and RNA-seq data. BMC bioinformatics. 2013;14:7.
15. Subramanian A, Tamayo P, Mootha VK, Mukherjee S, Ebert BL, Gillette MA, et al. Gene set enrichment analysis: a knowledge-based approach for interpreting genome-wide expression profiles. Proc Natl Acad Sci U S A. 2005;102(43):15545-50.
16. Li H, Durbin R. Fast and accurate short read alignment with Burrows-Wheeler transform. Bioinformatics. 2009;25(14):1754-60.
17. Zhang Y, Liu T, Meyer CA, Eeckhoute J, Johnson DS, Bernstein BE, et al. Model-based analysis of ChIP-Seq (MACS). Genome biology. 2008;9(9):R137.
18. McLean CY, Bristor D, Hiller M, Clarke SL, Schaar BT, Lowe CB, et al. GREAT improves functional interpretation of cis-regulatory regions. Nat Biotechnol. 2010;28(5):495-501.

19. Heinz S, Benner C, Spann N, Bertolino E, Lin YC, Laslo P, et al. Simple combinations of lineage-determining transcription factors prime cis-regulatory elements required for macrophage and B cell identities. Molecular cell. 2010;38(4):576-89.
20. Ramirez F, Ryan DP, Gruning B, Bhardwaj V, Kilpert F, Richter AS, et al. deepTools2: a next generation web server for deep-sequencing data analysis. Nucleic acids research. 2016;44(W1):W160-5.
21. Gu Z, Gu L, Eils R, Schlesner M, Brors B. circlize Implements and enhances circular visualization in R. Bioinformatics. 2014;30(19):2811-2.
22. Wei G, Abraham BJ, Yagi R, Jothi R, Cui K, Sharma S, et al. Genome-wide analyses of transcription factor GATA3-mediated gene regulation in distinct T cell types. Immunity. 2011;35(2):299-311.
23. Wang S, Sun H, Ma J, Zang C, Wang C, Wang J, et al. Target analysis by integration of transcriptome and ChIP-seq data with BETA. Nat Protoc. 2013;8(12):2502-15.

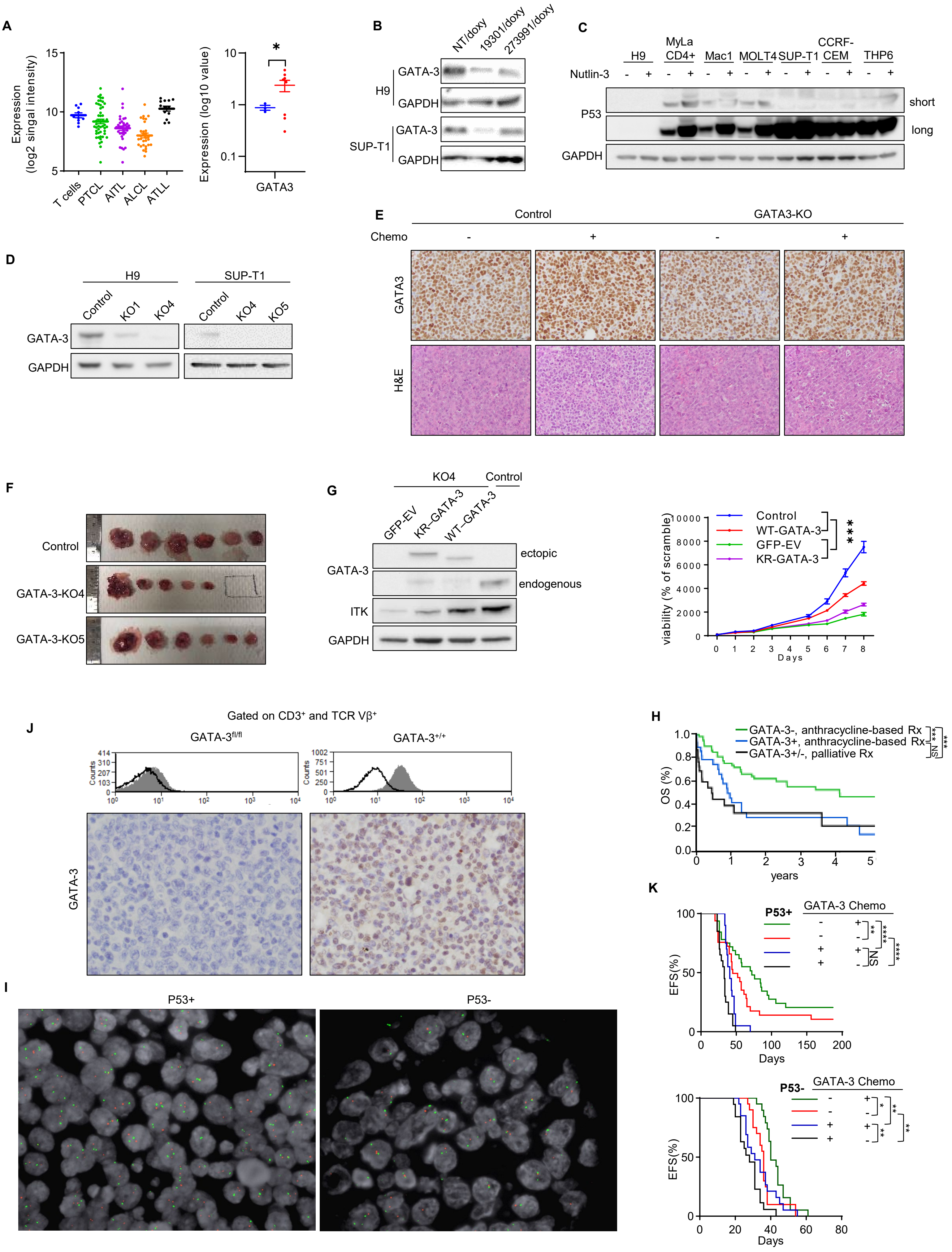
A
Expression (log2 singal intensity)
T cells
PTCL
AITL
ALCL
ATLL
Expression (log10 value)
GATA3
B
NT/doxy
19301/doxy
273991/doxy
H9
SUP-T1
GATA-3
GAPDH
C
H9
MyLa CD4+
Mac1
MOLT4
SUP-T1
CCRF-CEM
THP6
Nutlin-3
P53
short
long
GAPDH
D
H9
SUP-T1
Control
KO1
KO4
KO5
GATA-3
GAPDH
E
Control
GATA3-KO
Chemo
GATA3
H&E
F
Control
GATA-3-KO4
GATA-3-KO5
G
KO4
Control
GFP-EV
KR-GATA-3
WT-GATA-3
GATA-3
ectopic
endogenous
ITK
GAPDH
viability (% of scramble)
Control
WT-GATA-3
GFP-EV
KR-GATA-3
Days
H
GATA-3-, anthracycline-based Rx
GATA-3+, anthracycline-based Rx
GATA-3+/-, palliative Rx
OS (%)
years
J
Gated on CD3+ and TCR Vβ+
GATA-3fl/fl
GATA-3+/+
Counts
GATA-3
I
P53+
P53-
K
P53+
GATA-3 Chemo
EFS(%)
Days
P53-
GATA-3 Chemo
EFS(%)
Days

Supplemental Fig. S1 GATA-3 is a proto-oncogene in CTCL and T-ALL. **A,** GATA-3 transcript abundance from a publicly available cohort. Normal T cells, peripheral T cell lymphoma (PTCL), angioimmunoblastic T-cell Lymphoma (AITL), anaplastic large cell lymphoma (ALCL) and adult T-Cell Leukemia/Lymphoma (ATLL) were included (shown at left). GATA-3 expression was determined in normal T cells obtained from healthy donors (blue) and malignant T cells obtained from Sezary syndrome patients (red) and is shown (at right). **B,** GATA-3 expression was determined by immunoblot following doxycycline-inducible, shRNA-mediated GATA-3 knockdown with two independent GATA-3 targeting (19301 and 273991) and non-targeting (NT) shRNA. **C,** p53 expression in CTCL and T-ALL cell lines treated with and without the MDM2 inhibitor Nutlin-3 (5 μM, 16 h). Immunoblots after "short" and "long" exposure times are shown, as indicated. **D,** GATA-3 expression following GATA-3 knockout by CRISPR/Cas9. Control, cells with negative control sgRNA. **E,** GATA-3 expression was determined by immunohistochemistry in negative control gRNA (Control) and GATA-3 KO (KO4) H9 xenografts. In contrast to the cell lines generated (shown in c), tumor xenografts uniformly express GATA-3. **F,** Pictures of SUP-T1 xenografts generated in NSG mice bearing control (n=8) and GATA-3 KO xenografts (n=6). Control, cells with negative control sgRNA. **G,** GATA-3 expression (at left) and cell viability (at right) were determined by IB and RealTime-Glo, separately, on empty vector (GFP-EV), wildtype GATA-3 (WT-GATA-3), K358R/K377R mutated GATA-3 (KR-GATA-3) expressed in GATA-3 KO SUP-T1 cells. A corresponding western blot for GATA-3 (ectopic and endogenous) and a representative GATA-3 target gene (ITK) is show. Control, SUP-T1 cells with negative control sgRNA. **H,** A cohort of PTCL, NOS patients was stratified by GATA-3 expression and treatments received, and overall survival (OS) examined. **I,** Representative p53 FISH images obtained from PTCL, NOS biopsies. **J,** GATA-3 expression in $SNF5^{fl/fl}$, CD4-Cre $GATA\text{-}3^{fl/fl}$ and $SNF5^{fl/fl}$, CD4-Cre $GATA\text{-}3^{+/+}$ mice. Representative flow cytometry (top) and immuno-histochemical images (bottom) are shown. **K,** Splenocytes from lymphoma-bearing $GATA\text{-}3^{fl/fl}$ (n=13) or $GATA\text{-}3^{+/+}$ (n=80), $SNF5^{fl/fl}$, $p53^{+/+}$ CD4-Cre mice and $GATA\text{-}3^{fl/fl}$ (n=14) and $GATA\text{-}3^{+/+}$ (n=29) $SNF5^{fl/fl}$, $p53^{fl/fl}$, CD4-Cre mice were adoptively transferred to B6 recipients and treated with cyclophosphamide/vincritistine ("Chemo"), as inidicated, or vehicle control (n=4-5 recipient mice/group), and EFS determined. *$p < 0.05$; **$p < 0.01$, ***$p<0.001$ (Welch's unpaired t-test or log-rank test).

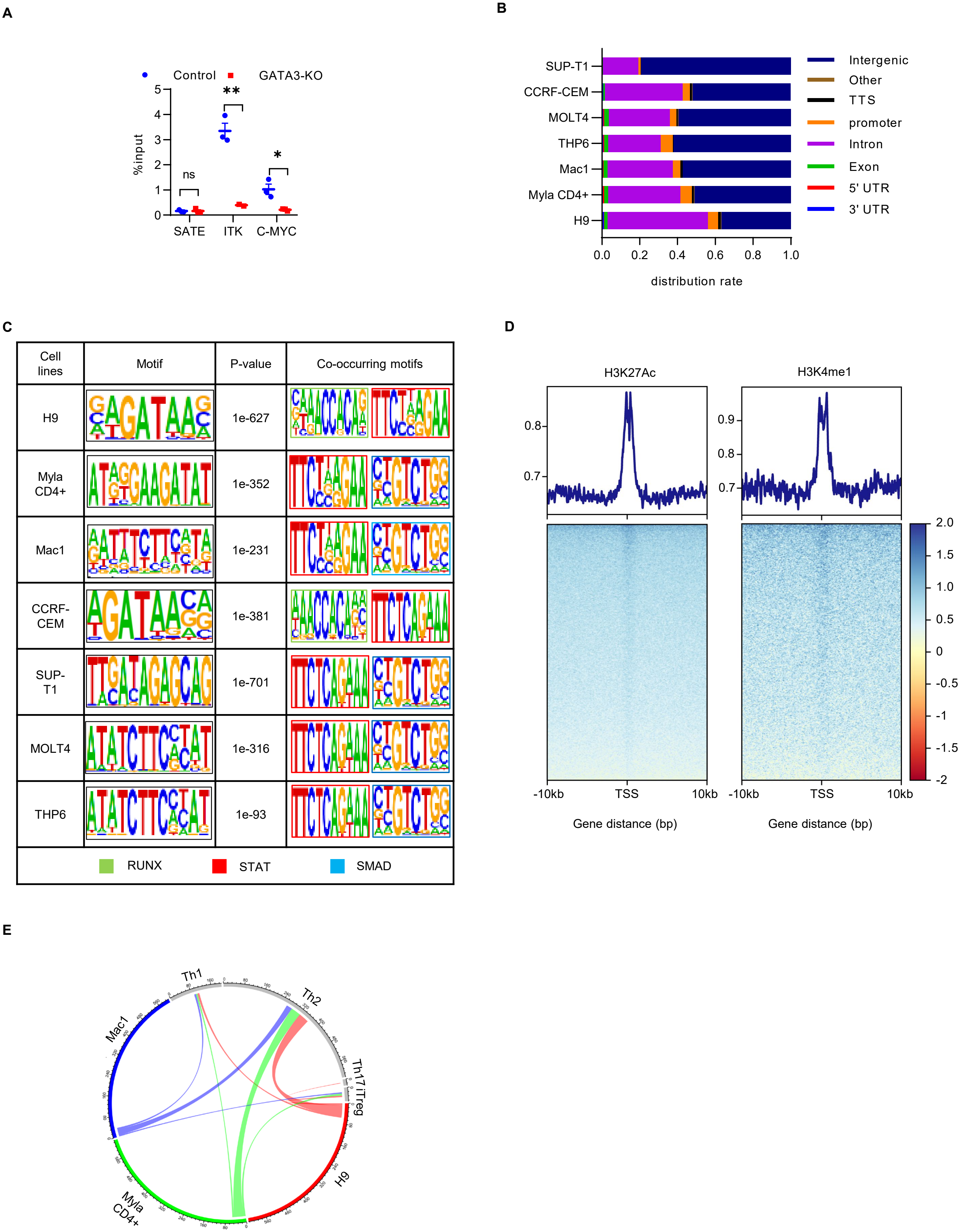
A
Control
GATA3-KO
%input
5
4
3
2
1
0
ns
**
*
SATE
ITK
C-MYC
B
SUP-T1
CCRF-CEM
MOLT4
THP6
Mac1
Myla CD4+
H9
0.0
0.2
0.4
0.6
0.8
1.0
distribution rate
Intergenic
Other
TTS
promoter
Intron
Exon
5' UTR
3' UTR
C
Cell lines
Motif
P-value
Co-occurring motifs
H9
1e-627
Myla CD4+
1e-352
Mac1
1e-231
CCRF-CEM
1e-381
SUP-T1
1e-701
MOLT4
1e-316
THP6
1e-93
RUNX
STAT
SMAD
D
H3K27Ac
H3K4me1
0.8
0.7
0.9
0.8
0.7
2.0
1.5
1.0
0.5
0
-0.5
-1
-1.5
-2
-10kb
TSS
10kb
-10kb
TSS
10kb
Gene distance (bp)
Gene distance (bp)
E
Th1
Th2
Th17
iTreg
Mac1
H9
Myla CD4+

Supplemental Fig. S2 GATA-3 target genes identified by integrated analysis of ChIP-seq and RNA-seq reveals transcriptional programs in CTCL and T-ALL. **A,** GATA-3 ChIP and binding enrichment at selected GATA-3 target genes (ITK, and C-MYC) is shown for the control and SUP-T1 KO cells. SATE, human α satellite repeat element. **B,** Distribution of GATA-3 binding peaks within the genome. TTS, transcription termination site. C, Motif enrichment analysis of GATA-3 binding sites in CTCL and T-ALL. Top-ranked co-occurring binding motifs are also displayed. **D,** H3K4me1 and H3K27Ac distribution near GATA-3 binding peaks in H9 cells. Upper panels show the average signal profile on genomic loci defined as 10kb upstream and downstream of transcript start site (TSS). Lower panels show density heatmap around the same genomic loci. **E,** Chord diagram showing relations between GATA-3 target genes in CTCL cell lines and normal T helper cell subsets.

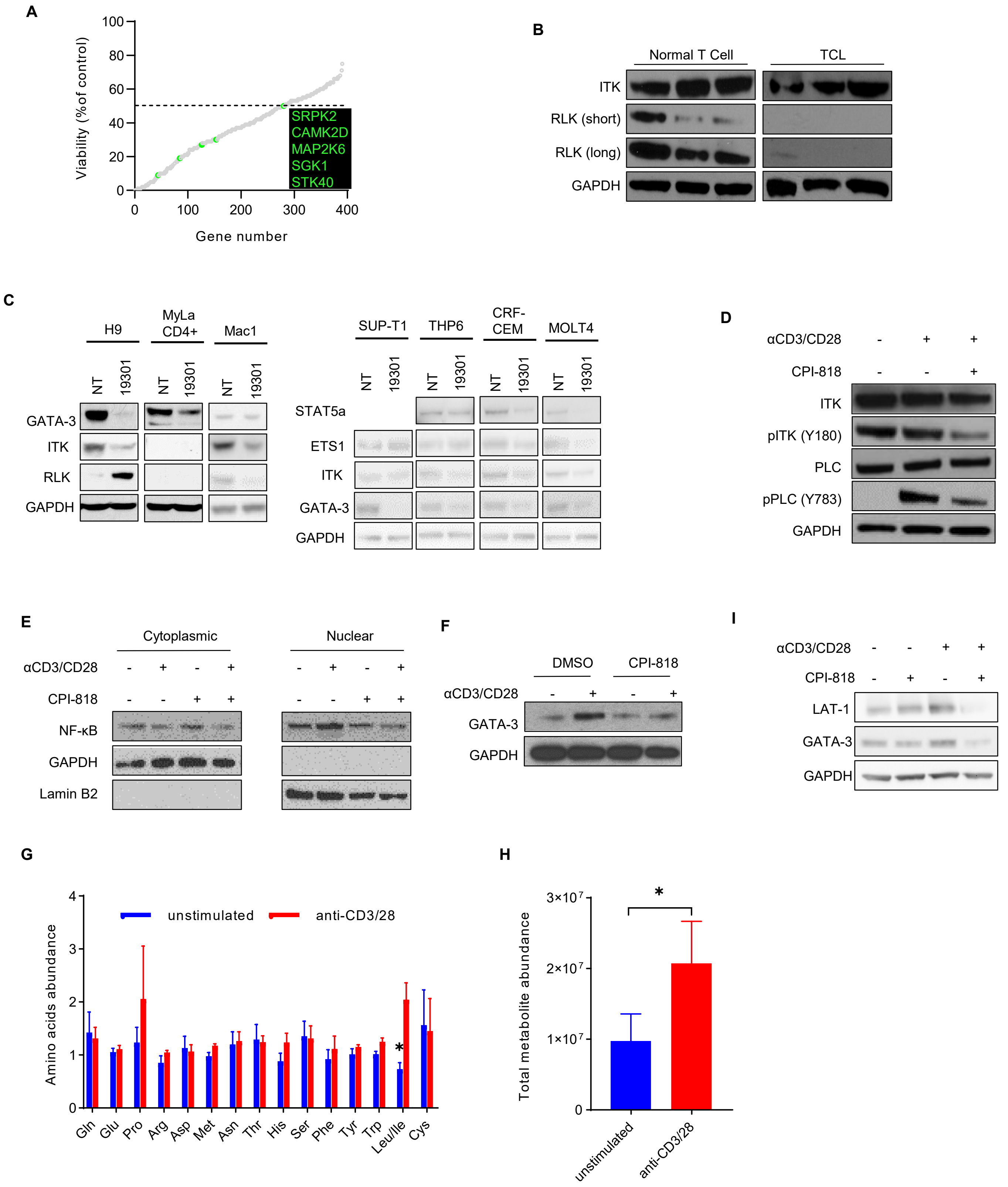

A
Viability (%of control)
Gene number
SRPK2
CAMK2D
MAP2K6
SGK1
STK40
B
Normal T Cell
TCL
ITK
RLK (short)
RLK (long)
GAPDH
C
H9
MyLa CD4+
Mac1
NT
19301
GATA-3
ITK
RLK
GAPDH
SUP-T1
THP6
CRF-CEM
MOLT4
STAT5a
ETS1
ITK
GATA-3
GAPDH
D
αCD3/CD28
CPI-818
ITK
pITK (Y180)
PLC
pPLC (Y783)
GAPDH
E
Cytoplasmic
Nuclear
αCD3/CD28
CPI-818
NF-κB
GAPDH
Lamin B2
F
DMSO
CPI-818
αCD3/CD28
GATA-3
GAPDH
I
αCD3/CD28
CPI-818
LAT-1
GATA-3
GAPDH
G
Amino acids abundance
unstimulated
anti-CD3/28
Gln Glu Pro Arg Asp Met Asn Thr His Ser Phe Tyr Trp Leu/Ile Cys
H
Total metabolite abundance
unstimulated
anti-CD3/28

Supplemental Fig. S3 | ITK as a therapeutic vulnerability. A, A shRNA-based loss-of-function viability screen was performed using shRNA targeting ≈500 tyrosine kinases in MyLa-CD4+ cells, and previously published. GATA-3 dependent kinases associated with ≥50% reduction in viability upon knockdown are highlighted, and listed in the inset. **B,** Immunoblot analysis of ITK and RLK expression in the normal T cells and malignant T cells obtained from a patient with Sezary syndrome. **C,** Immunoblot analysis of indicated protein expression levels in GATA-3 knockdown cells. **D-E,** Jurkat cells were treated with various combinations of anti-CD3/CD28 beads and CPI-818 (1 μM) and ITK (Y180) and PLCγ (Y783) phosphorylation (D) and NF-kB (p65) nuclear localization (E) determined by immunoblotting. **F,** Jurkat were similarly treated and GATA-3 expression determined 24 hours later by immunoblotting. **G-H,** Amino acid (G) and total metabolite (H) abundance was determined by mass spectrometry in primary T-cell lymphoma specimens cultured for 24 hours in the presence or absence of anti-CD3/CD28 beads. **I,** T8ML-1 cells were similarly treated with anti-CD3/CD28 beads in the presence of CPI-818 (1 μM) or vehicle control, as indicated, and GATA-3 and LAT-1 expression determined.

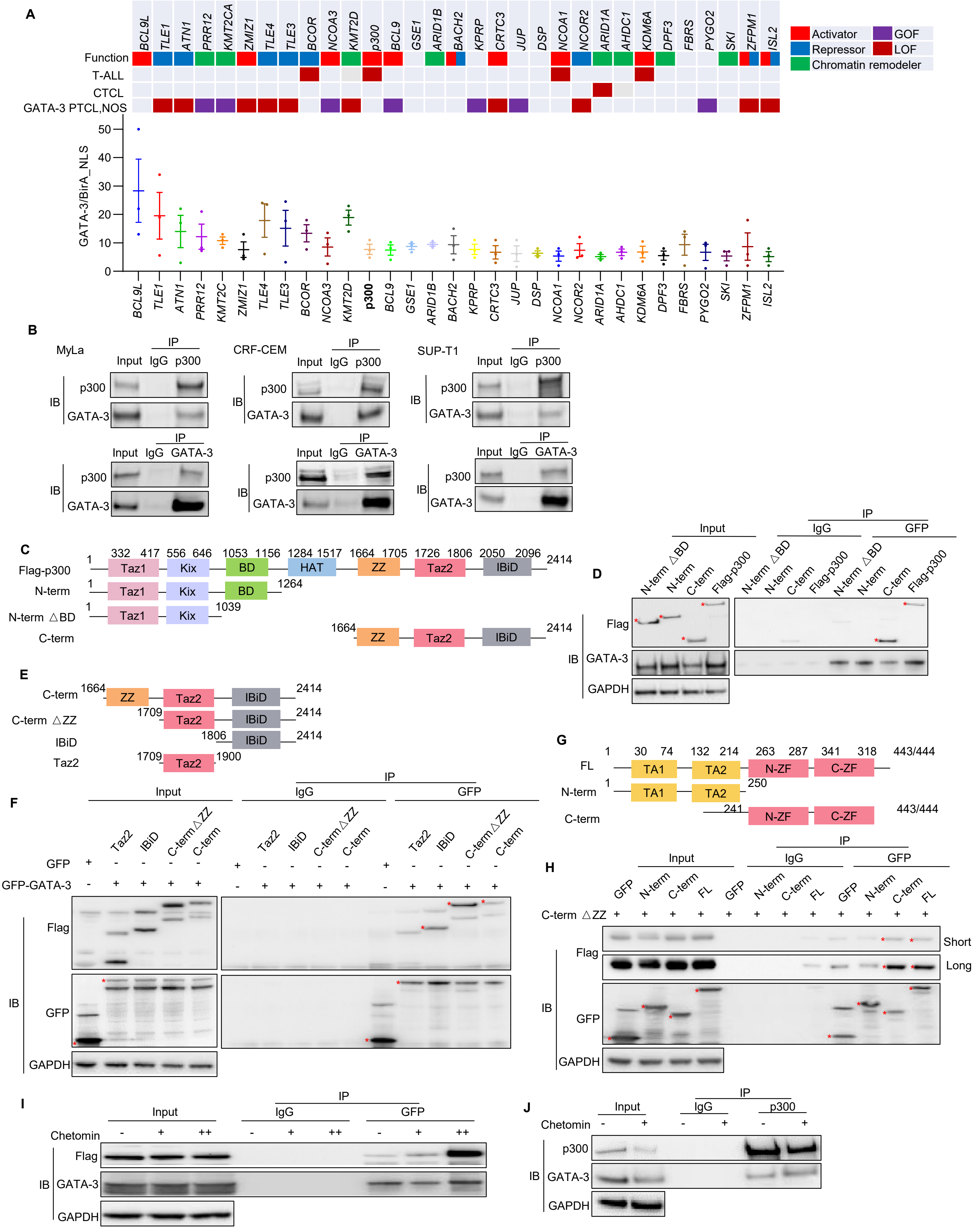

A
Function
T-ALL
CTCL
GATA-3 PTCL,NOS
Activator
Repressor
Chromatin remodeler
GOF
LOF
GATA-3/BirA_NLS
BCL9L TLE1 ATN1 PRR12 KMT2CA ZMIZ1 TLE4 TLE3 BCOR NCOA3 KMT2D p300 BCL9 GSE1 ARID1B BACH2 KPRP CRTC3 JUP DSP NCOA1 NCOR2 ARID1A AHDC1 KDM6A DPF3 FBRS PYGO2 SKI ZFPM1 ISL2
B
MyLa
CRF-CEM
SUP-T1
IP
Input IgG p300
Input IgG GATA-3
IB
p300
GATA-3
C
Flag-p300
N-term
N-term △BD
C-term
Taz1 Kix BD HAT ZZ Taz2 IBiD
D
Input
IgG
GFP
N-term △BD
N-term
C-term
Flag-p300
Flag
GAPDH
E
C-term △ZZ
IBiD
Taz2
F
GFP
GFP-GATA-3
G
FL
TA1 TA2 N-ZF C-ZF
H
Short
Long
FL
I
Chetomin
J

Supplemental Fig. S4 | P300 binds GATA-3 independently from the p300 bromodomain. A, GATA-3-binding partners identified by Bio-ID in Jurkat cells (n=3 independent experiments) are summarized (bottom) and their function and type of genetic alterations in T-ALL, CTCL, and GATA-3 PTCL, NOS summarized (top). **B,** Co-Immunoprecipitation (Co-IP)/immunoblotting (IB) in MyLa, CRF-CEM and SUP-T1 cells with IgG control antibodies or anti-P300 or anti-GATA-3 antibodies. **C, E, G,** Schematic of P300 (C, E) and GATA-3 (G) constructs used in the IP. **D, F, H,** IP in HEK293T cells transfected with indicated flag-tagged P300 and GFP-tagged GATA-3 (D, F) or indicated GFP- tagged GATA-3 and flag-tagged C-term △ZZ P300 (H) with IgG control antibodies or anti-GFP antibodies. Asterisks indicate the protein band. **I,** IP in HEK293T cells transfected with C-term P300 and GFP-GATA-3 with IgG control antibodies or anti-GFP antibodies after 1 μM P300 TAZ domain inhibitor (Chetomin) for 6 hours. **J,** IP in H9 cells with IgG control antibodies or anti-P300 antibodies after 1 μM Chetomin treatment for 6 hours.

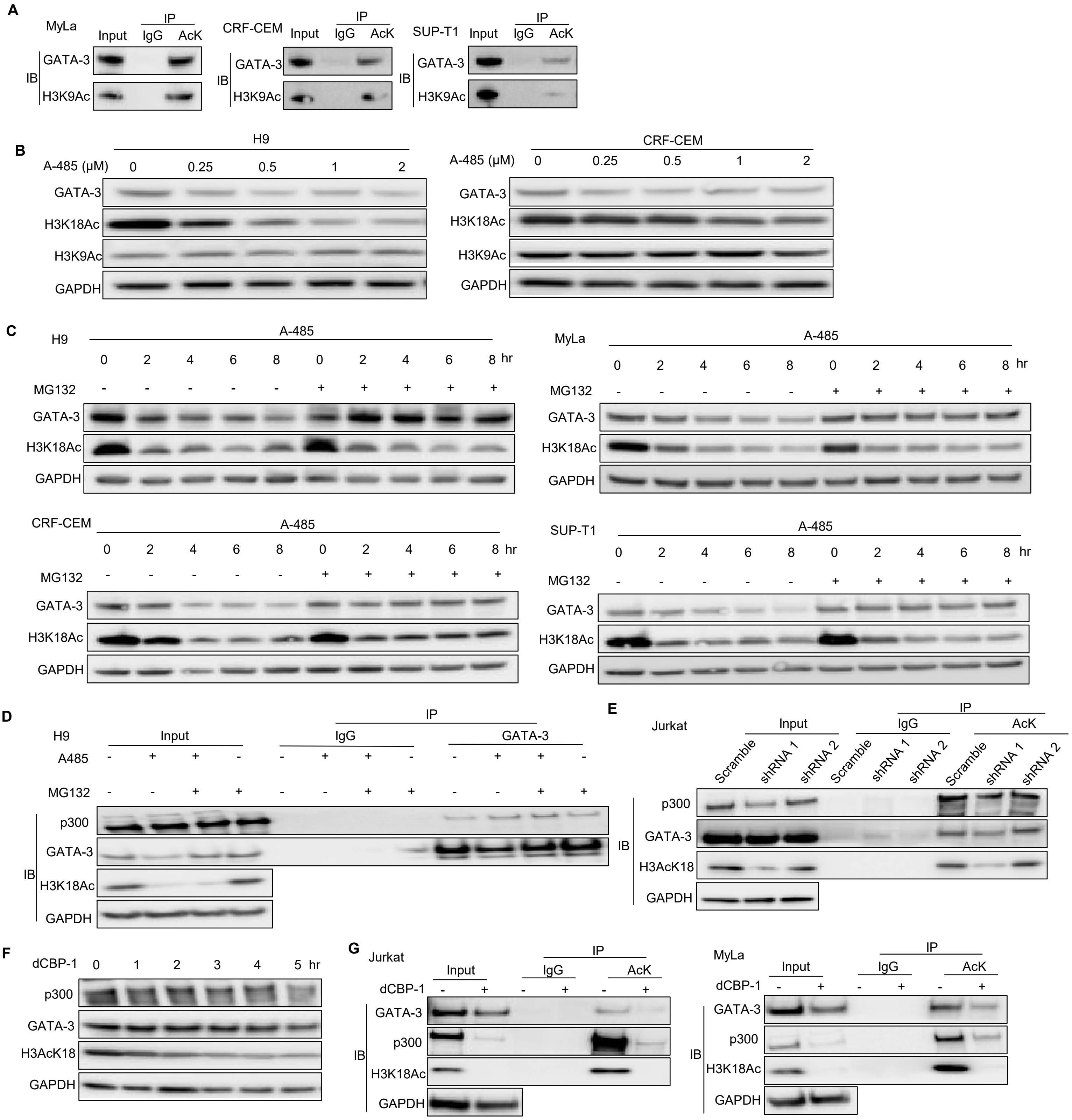
A
MyLa
IP
Input
IgG
AcK
IB
GATA-3
H3K9Ac
CRF-CEM
SUP-T1
B
H9
A-485 (μM)
0
0.25
0.5
1
2
GATA-3
H3K18Ac
H3K9Ac
GAPDH
CRF-CEM
C
H9
A-485
0
2
4
6
8
hr
MG132
GATA-3
H3K18Ac
GAPDH
MyLa
CRF-CEM
SUP-T1
D
H9
IP
Input
IgG
GATA-3
A485
MG132
IB
p300
GATA-3
H3K18Ac
GAPDH
E
Jurkat
Input
IP
IgG
AcK
Scramble
shRNA 1
shRNA 2
IB
p300
GATA-3
H3AcK18
GAPDH
F
dCBP-1
0
1
2
3
4
5
hr
p300
GATA-3
H3AcK18
GAPDH
G
Jurkat
IP
Input
IgG
AcK
dCBP-1
IB
GATA-3
p300
H3K18Ac
GAPDH
MyLa

Supplemental Fig. S5 | P300 acetylase GATA-3. **A,** Immunoprecipitation (IP) in MyLa, CRF-CEM and SUP-T1 cells with IgG control antibodies or anti-acetyl lysine antibodies and immunoblotting (IB) with GATA-3 or H3K9Ac antibodies. **B-C,** Immunoblot analysis of GATA-3 expression in cells treated with A485 alone (B) or with 5 μM MG132 (C) for 6 hours. **D,** IP in H9 cells with IgG control antibodies or anti-GATA-3 antibodies after 2 μM A485 alone or with 5 μM MG132 for 6 hours. **E,** IP in Jurkat cells transduced with scramble shRNA or P300 targeting shRNA with IgG control antibodies or anti-acetyl lysine antibodies, followed by immunoblotting (IB) for GATA-3 and H3K18Ac. Of note, only shRNA1 led to modest p300 knockdown. **F,** Immunoblot analysis of GATA-3 expression in H9 cells treated with 1 μM dCBP-1 for the times indicated. **G,** IP in Jurkat (left) and MyLa (right) cells with IgG control antibodies or anti-acetyl lysine antibodies after 1 μM dCBP-1 treatment for 5 hours.

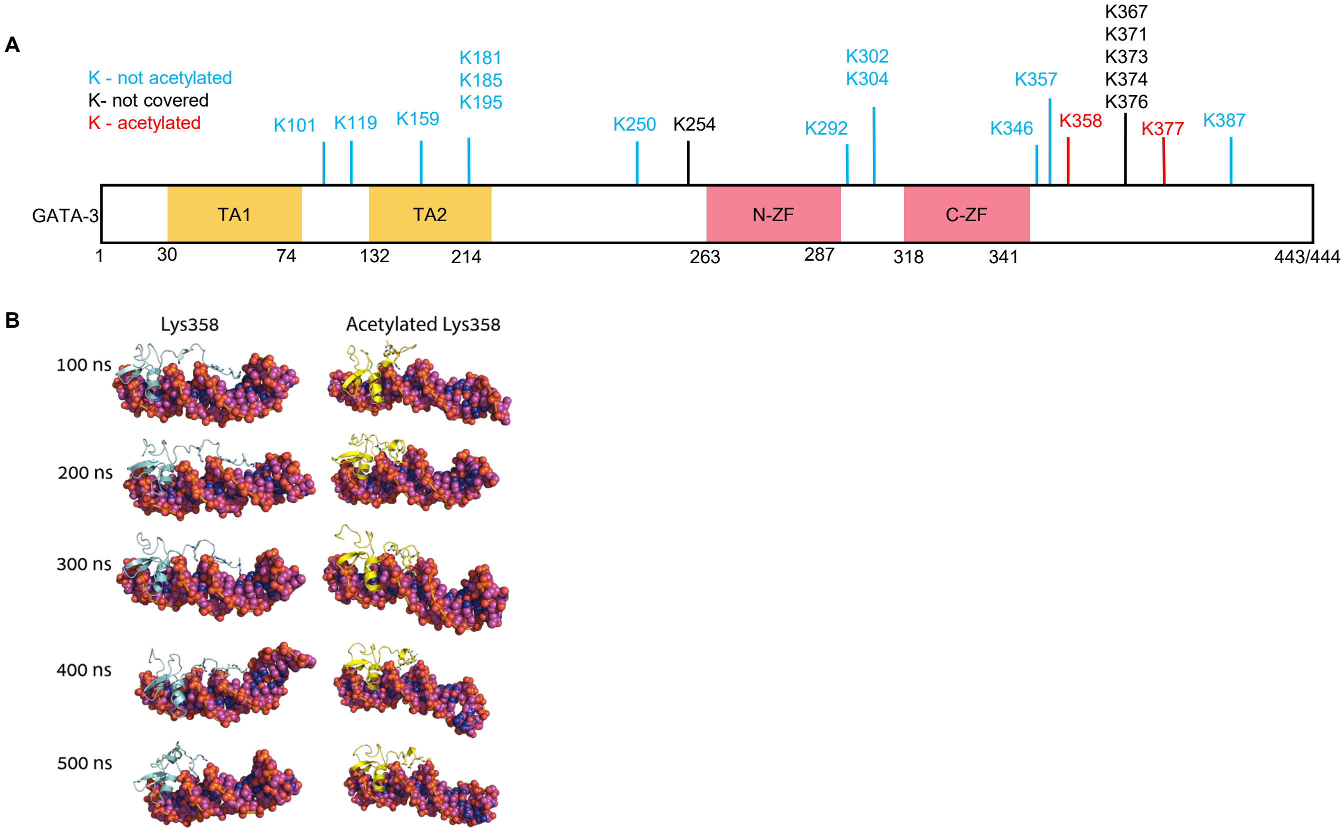
A
K - not acetylated
K- not covered
K - acetylated
K101
K119
K159
K181
K185
K195
K250
K254
K292
K302
K304
K346
K357
K358
K367
K371
K373
K374
K376
K377
K387
GATA-3
TA1
TA2
N-ZF
C-ZF
1
30
74
132
214
263
287
318
341
443/444
B
Lys358
Acetylated Lys358
100 ns
200 ns
300 ns
400 ns
500 ns

Supplemental Fig. S6 | GATA-3 is acetylated at K358 and K377. A, HEK293T cells were transfected with GFP-tagged GATA-3 and GATA-3 immunoprecipitated. Mass spectrometry was performed and the data summarized here. GATA-3 has 21 lysine residues, 15 of which were covered by mass spectrometry (shown in blue). Two lysines (K358, K377) were acetylated. **B,** Still shots at time points indicated of molecular dynamics simulations performed in GATA-3 unacetylated at K358 (left) or acetylated at K358 (right).

CTCL

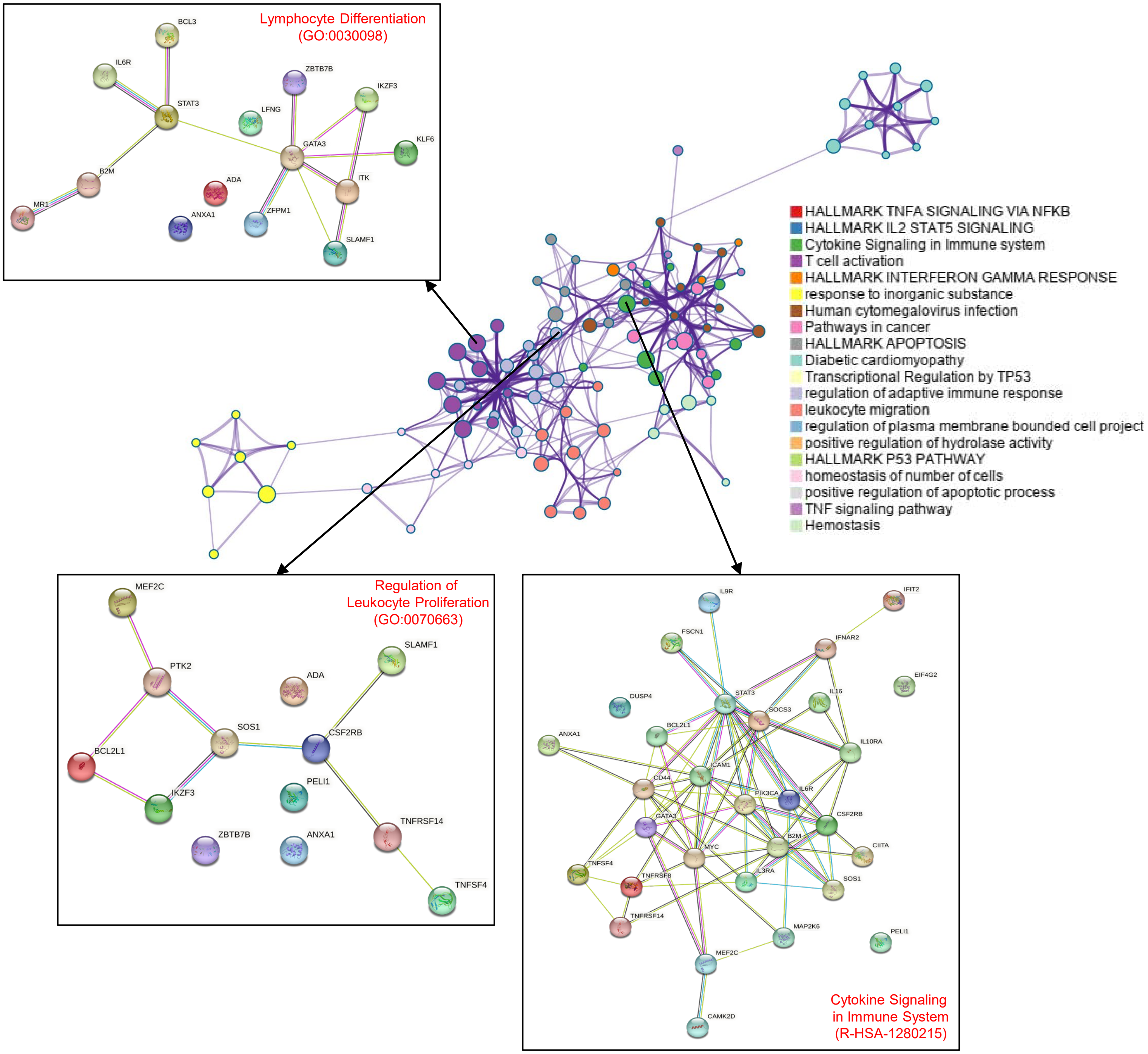
Lymphocyte Differentiation
(GO:0030098)
BCL3
IL6R
ZBTB7B
IKZF3
STAT3
LFNG
KLF6
GATA3
B2M
ADA
ITK
MR1
ANXA1
ZFPM1
SLAMF1
HALLMARK TNFA SIGNALING VIA NFKB
HALLMARK IL2 STAT5 SIGNALING
Cytokine Signaling in Immune system
T cell activation
HALLMARK INTERFERON GAMMA RESPONSE
response to inorganic substance
Human cytomegalovirus infection
Pathways in cancer
HALLMARK APOPTOSIS
Diabetic cardiomyopathy
Transcriptional Regulation by TP53
regulation of adaptive immune response
leukocyte migration
regulation of plasma membrane bounded cell project
positive regulation of hydrolase activity
HALLMARK P53 PATHWAY
homeostasis of number of cells
positive regulation of apoptotic process
TNF signaling pathway
Hemostasis
Regulation of
Leukocyte Proliferation
(GO:0070663)
MEF2C
PTK2
ADA
SLAMF1
SOS1
CSF2RB
BCL2L1
PELI1
IKZF3
TNFRSF14
ZBTB7B
ANXA1
TNFSF4
IL9R
IFIT2
FSCN1
IFNAR2
EIF4G2
DUSP4
STAT3
IL16
SOCS3
BCL2L1
ANXA1
IL10RA
ICAM1
CD44
PIK3CA
IL6R
GATA3
CSF2RB
B2M
CIITA
MYC
TNFSF4
IL3RA
TNFRSF8
SOS1
TNFRSF14
MAP2K6
PELI1
MEF2C
CAMK2D
Cytokine Signaling
in Immune System
(R-HSA-1280215)

Supplemental Figure S7| Description of GATA-3 target genes in CTCL and their interaction. Pathways enrichment were analyzed on CTCL GATA-3 target genes separately. A subset of enriched terms have been selected and rendered as a network plot, where terms with a similarity > 0.3 are connected by edges and is visualized by Cytoscape. Each node represents an enriched pathway and is colored by their categories, which is shown on the right of network. Protein-protein interaction (PPI) were also analyzed and zoomed in on some represented pathways according to their physical score (>0.132) determined by STRING. In the PPI analysis, indigo and purple line were known interactions from curated databases and experimentally database, respectively. Green, red and blue lines are predicted interactions. Gene names were shown on each node.

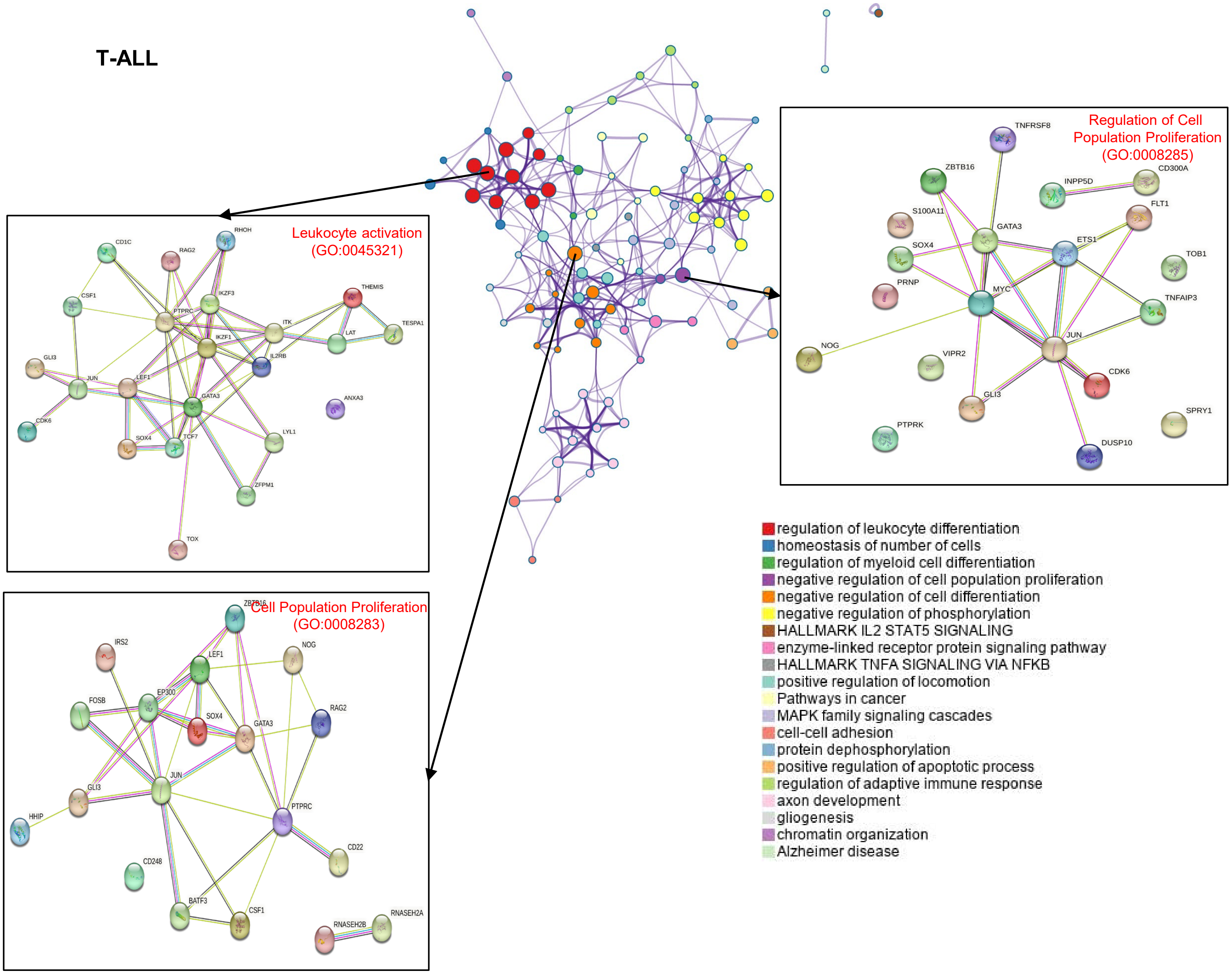
T-ALL
Leukocyte activation
(GO:0045321)
Cell Population Proliferation
(GO:0008283)
Regulation of Cell
Population Proliferation
(GO:0008285)
regulation of leukocyte differentiation
homeostasis of number of cells
regulation of myeloid cell differentiation
negative regulation of cell population proliferation
negative regulation of cell differentiation
negative regulation of phosphorylation
HALLMARK IL2 STAT5 SIGNALING
enzyme-linked receptor protein signaling pathway
HALLMARK TNFA SIGNALING VIA NFKB
positive regulation of locomotion
Pathways in cancer
MAPK family signaling cascades
cell-cell adhesion
protein dephosphorylation
positive regulation of apoptotic process
regulation of adaptive immune response
axon development
gliogenesis
chromatin organization
Alzheimer disease

Supplemental Figure S8| Description of GATA-3 target genes in T-ALL and their interaction. Pathways enrichment were analyzed on T-ALL GATA-3 target genes separately. A subset of enriched terms have been selected and rendered as a network plot, where terms with a similarity > 0.3 are connected by edges and is visualized by Cytoscape. Each node represents an enriched pathway and is colored by their categories, which is shown on the right of network. Protein-protein interaction (PPI) were also analyzed and zoomed in on some represented pathways according to their physical score (>0.132) determined by STRING. In the PPI analysis, indigo and purple line were known interactions from curated databases and experimentally database, respectively. Green, red and blue lines are predicted interactions. Gene names were shown on each node.

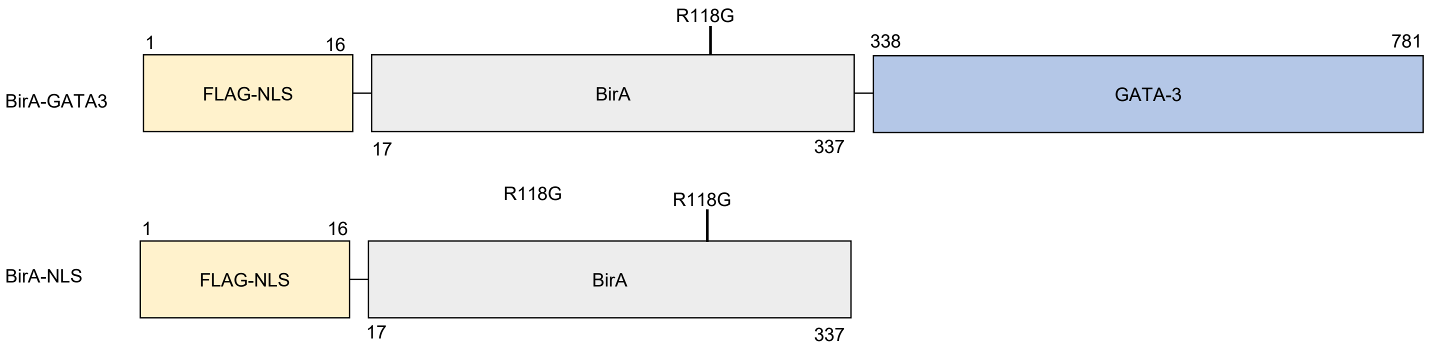
BirA-GATA3
R118G
1
16
FLAG-NLS
BirA
17
337
338
781
GATA-3
BirA-NLS
R118G
R118G
1
16
FLAG-NLS
BirA
17
337

Supplemental Figure S9| GATA-3 construct used in the BIO-ID Assay. Schematic representation of the E. Coli biotin ligase which harbors an R118G mutation (BirA) fused with full-length human GATA-3 (upper panel). The negative control vector contained a FLAG-tagged sequence with a nuclear localization signal (NLS) (lower panel).

Supplementary Table 1. Patient characteristics in PTCL, NOS cohorts

**Histopathologic characteristics**	**GATA-3 negative, treated (n=61)**	**GATA-3 positive, treated (n=28)**	**GATA-3 neg. or pos., best supportive care (n=33)**
GATA-3+	0/61 (0%)	28/28 (100%)	23/29 (79%)
GATA-3 H-score, mean (range)	4.3 (0-40)	139.3 (50-300)	36.9 (0-240)
**p53 deletion (FISH)**			
Normal	31/40 (78%)	11/14 (79%)	14/17 (82%)
Deleted	4/40 (10%)	3/14 (21%)	3/17 (18%)
Polyploidy +/- Deletion	5/40 (13%)	0/14	0/17
**Clinical characteristics**			
Age >60 (%)	30/61 (49%)	14/28 (50%)	23/33 (70%)
ECOG performance status >1 (%)	17/54 (31%)	11/27 (41%)	21/30 (70%)
LDH >normal (%)	35/56 (63%)	18/25 (72%)	21/28 (75%)
>1 site of extranodal disease (%)	21/61 (38%)	10/27 (37%)	5/27 (19%)
Ann Arbor Stage III/IV (%)	51/60 (85%)	24/28 (86%)	23/29 (79%)
**Frontline Treatment**			
CHOP or CHOEP	53/61 (87%)	24/28 (86%)	N/A
Alternative anthracycline-based regimen	8/61 (13%)	4/28 (14%)	N/A
Non-anthracycline-based regimen or single-agent	N/A	N/A	6/33 (18%)
Palliative radiation	N/A	N/A	4/33 (12%)
Supportive care/hospice +/- corticosteroids	N/A	N/A	23/33 (72%)
**Response to Frontline Treatment**	**n=58 (95%)**	**n=26 (93%)**	**n=13 (39%)**
CR	26/58 (45%)	8/26 (31%)	1/13 (8%)
PR	16/58 (28%)	4/26 (15%)	4/13 (31%)
Stable	1/58 (2%)	1/26 (4%)	0
Progression	15/58 (26%)	13/26 (50%)	8/13 (62%)
**1st Salvage Treatment**	**n=32 (52%)**	**n=16 (57%)**	**n=5 (15%)**
ICE	8/32 (28%)	2/16 (13%)	0
DHAP	3/32 (10%)	0	0
Romidepsin	2/32 (7%)	6/16 (38%)	0
Brentuximab vedotin	2/32 (7%)	1/16 (6%)	0
Pralatrexate	1/32 (3%)	0	0
Other salvage regimen +/- radiation	7/32 (22%)	4/16 (25%)	4/5 (80%)
Clinical trial	3/32 (9%)	3/16 (19%)	0
BSC	1/32 (3%)	1/16 (6%)	0
Unknown	0	0	1/5 (20%)
**Response to Salvage Treatment**	**n=27**	**n=14**	**n=5**
CR	9/27 (33%)	1/14 (7%)	1/5 (20%)
PR	5/27 (19%)	3/14 (21%)	0
Stable	2/27 (7%)	0	1/5 (20%)
Progression	11/27 (41%)	10/14 (71%)	3 (60%)

Last Treatment Among Those Achieving EFS24	n=18	n=2	n=2
Firstline Therapy	14/18 (78%)	1/2 (50%)	
Pralatrexate	2/18 (11%)		
ICE	1/18 (6%)		
GIFOX			1/2 (50%)
Vorinostat			1/2 (50%)
PD-1 Blockade (Clinical Trial)	1/18 (6%)	1/2 (50%)	

Supplementary Table S2. Mature T-cell Lymphoma Patient Characteristics (ex vivo studies

Patient ID	Diagnosis	Notes
#1	Sezary Syndrome	CD3+CD4+CD26-CD7- (89% of total lymphocytes)
#2	Sezary Syndrome	CD3+CD4+CD26-CD7- (86% of total lymphocytes)
#3	Sezary Syndrome	CD3+CD4+CD26-CD7- (94%% of CD4+ T cells)
#4	Sezary Syndrome	CD3+CD4+CD26-CD7- (85% of total lymphocytes)
#5	PTCL, NOS	Stage IVB, including secondary leukemic involvement, representing 95% of total lymphocytes
#6	Sezary Syndrome	CD3+CD4+CD26-CD7- (95% of total lymphocytes)
#7	Sezary Syndrome	$CD3^{dim}$CD4+CD26-CD7- (91% of total lymphocytes)
#8	Hepatosplenic T-cell lymphoma	Stage IVA, CD3+CD4-$CD8^{dim}$CD7-TCRαβ-, TCRγδ+ (71% of spleen cellularity)

Supplementary Table 3. Primers and Sequences

Name	Sequence	Application
hGATA-3 K358R Mut F	ACCCCTGACTATGAAGAGGGAAGGCATCCAGACCA	Mutagenesis
hGATA-3 K358R Mut R	TGGTCTGGATGCCTTCCCTCTTCATAGTCAGGGGT	
hGATA-3 K377R Mut F	ATCCAAAAAGTGCAAAAGAGTGCATGACTCACTGG	
hGATA-3 K377R Mut R	CCAGTGAGTCATGCACTCTTTTGCACTTTTTGGAT	
hGATA-3 K358Q Mut F	GACCCCTGACTATGAAGCAGGAAGGCATCCAGACC	
hGATA-3 K358Q Mut R	GGTCTGGATGCCTTCCTGCTTCATAGTCAGGGGTC	
hGATA-3 K377Q Mut F	AATCCAAAAAGTGCAAACAAGTGCATGACTCACTG	
hGATA-3 K377Q Mut R	CAGTGAGTCATGCACTTGTTTGCACTTTTTGGATT	
p300 C-term (XhoI) – Flag (1664) F	TTCCTTCTCGAGATGGACTACAAAGACGATGACGACAAGGACCGCTTTGTCTACAC	PCR p300 (1664-2414aa)
p300 C-term △ZZ (XhoI) – Flag (1709) F	TTCCTTCTCGAGATGGACTACAAAGACGATGACGACAAGGGCCTTGGCTTAGATGAT	PCR p300 (1709-2414aa)
p300 IBiD (XhoI) – Flag (1806) F	TTCCTTCTCGAGATGGACTACAAAGACGATGACGACAAGTGCCTAAACATCAAG	PCR p300 (1806-2414aa)
p300 Taz2 (BamHI)– (1900) R	TTCCTTGGATCCCTACTTACCCTGGGACACA	PCR p300 (1709-1900aa)
p300 (BamHI)– (2414) R	TTCCTTGGATCCCTAGTGTATGTCTAGTG	PCR p300
GATA-3 (XhoI) – (1) F	TTCCTTCTCGAGCTATGGAGGTGACGGCGGACC	PCR GATA-3 (1-250aa)
GATA-3 (EcoRI)- (250) R	TTCCTTGAATTCCTACATCACCATCACCATCACCTTGCATCCGAAGCCGGTG	PCR GATA-3 (1-250aa)
GATA-3 (XhoI) – (241) F	TTCCTTCTCGAGCTATGGGCGGCTCCCCCACCGGCT	PCR GATA-3 (241-444aa)
GATA-3 (EcoRI)- (444) R	TTCCTTGAATTCCTACATCACCATCACCATCACACCCATGGCGGTGACCATG	PCR GATA-3
hGATA-3 promoter (XhoI) F	TTCCTTCTCGAGGGGCGGGGGGAGAAGTCCT	PCR GATA-3 promoter region (−148/+587)
hGATA-3 promoter (EcoRV) R	TTCCTTGATATCGAATGGGAGACCTGGAT	
GATA-3 DNA-binding assay WT probe	AGAATGTAGCCCTGGACTTCTCCCGCTCGCTATCAGATAAGGCCTTATUUCGATAAGGCCTTATCTGATAGCGAGCGGGAGAAGTCCAGGGCTACATTCT	GATA-3 DNA-binding assay
GATA-3 DNA-binding assay Mut probe	AGAATGTAGCCCTGGACTTCTCCCGCTCGCAAAAATTTTAGGCCTTATUUCGATAAGGCCTAAA	

	ATTTTTGCGAGCGGGAGAAGTCCAGGGCTAC ATTCT	
hGATA-3 DNA-bind qPCR F<br>hGATA-3 DNA-bind qPCR R	AGAATGTAGCCCTGGACTTC<br><br>TTCTCCCGCTCGCTATCA	GATA-3 DNA-binding assay qPCR detection
Satellite (SATE) Primers<br>ITK-F<br>ITK-R<br>CCR4-F<br>CCR4-R<br>C-MYC-F<br>C-MYC-R<br>IKZF3-F<br>IKZF3-R<br>ZFPM1-F<br>ZFPM1-R<br>HES1-F<br>HES1-R<br>IL2-F<br>IL2-R<br>TCF7-F<br>TCF7-R<br>PIM-F<br>PIM-R<br>NUAK1-F<br>NUAK1-R<br>LEF-F<br>LEF-R	#4486 (CST)<br><br>GCCTCCTCGTTTTGTGAATTTT<br>GATGTCAAAGGCCGAAACCA<br>AAGAAAGATCCTTATCAGACAGAAAGA<br>TTTCCACCTAGTGTCAGATACCT<br>GGGTAATTGCAGAGGAAGGATTAG<br>GTGACGGTGTATGATCAGTGAAAG<br>GGTAACTTGAGCAGTGCAGATG<br>CTGTAGTTTACCCTTTCTGGGTTTC<br>TTGTTCCGTGCGGAGATAGA<br>CGCTCCCACGTTTGAAGTTAATA<br>CTTCAATGATGTTGCCTCCCT<br>CAGTTTGGAGTCGTGCCTTAT<br>TGGTTAGTCAGTAAATACAGGTCTACT<br>CCCAACCCAATCTTCCTTTCAG<br>GAGCGTTATCTGCACCATCAAA<br>CTCTTCCTTTCAACAGTCAAACCT<br>AAGTCTTGATGCTAGGGAAACAAA<br>GTTTCAGAACCCATGGTCTGTC<br>TGGGAGTTGGACGTGTCTG<br>GAAACCTTCAGTTGGGCAGATTA<br>CAGGTGCTTTGCAAGAGATGA<br>AGAAAGAGGTGCTATGACAAACTG	ChIP-qPCR
CCR4-F<br>CCR4-R<br>C-MYC-F<br>C-MYC-R<br>GAPDH-F<br>GAPDH-R<br>HPRT1-F<br>HPRT1-R<br>ITK-F<br>ITK-R<br>IKZF3-F<br>IKZF3-R<br>HES1-F<br>HES1-R<br>IL2-F<br>IL2-R<br>ZFPM1-F<br>ZFPM1-R<br>TCF7-F	CTCTGGCTTTTGTTCACTGCTGC<br>AGCCCACAGTATTGGCAGAGCA<br>CACATCAGCACAACTACGCA<br>GGTGCATTTTCGGTTGTTGC<br>TCTGACTTCAACAGCGACAC<br>TGTCATACCAGGAAATGAGCTT<br>GTGAAAAGGACCCCACGAAG<br>TCCAAACTCAACTTGAACTCTCA<br>GCCCAAAGTGATGGAACTAGAA<br>CAGTAGAAGCCAGTGTCGAATAG<br>GCCTGAAATCCCTTACAGCTATTC<br>ACTGGTTGGCCTGCTACTATC<br>GTCAACACGACACCGGATAAAC<br>CAGCTGGCTCAGACTTTCATTT<br>AACTCACCAGGATGCTCACATTTA<br>TCAGTTCTGTGGCCTTCTTGG<br>CCTCAAACTCCTGAGCTGAAAC<br>CACAGGAAGACCCTGTCTCTATAA<br>CAGAGCCTGTCTTTCAGCATTC	qRT-PCR

TCF7-R<br>PIM1-F<br>PIM1-R	CTGGGCTCCTTGATCTAGTTGTA<br>GAAACAGGTTGGGATGGGATAG<br>CACAGAAGGCACTCAGAAAGAG	
NT<br>19301 (TRCN0000019301)<br>273991 (TRCN0000273991)	GGCGCGATAGCGCTAATAATTT<br>CATCCAGACCAGAAACCGAAA<br>AGCCTAAACGCGATGGATATA	shRNA knock-down
EP300 (TRCN0000231133)	TAACCAATGGTGGTGATATTA	
EP300 (TRCN0000231134)	ATACTCAGCCGGAGGATATTT	
Hs.Cas9.GATA3.1.AB	GGAGCTGTACTCGGGCACGT	CRISPR
SNF5-F<br>SNF5-R<br>CD4-Cre-F<br>CD4-Cre-R	CACCATGCCCCCACCTCCCCTACA<br>CAGGAAAATGGATGCAACTAAGAT<br>TTCCCAACCAACAAGAGCTC<br>GGACCGACGATGAAGCATGT	Genotyping

Supplementary Table 4. Antibodies

Antigen	Clone(s)	Flourochrome(s)	Source (cat. #)	Additional information
Acetylated-Lysine	Polyclonal		#9441 (CST)	IP: 1:100
Flag	Monoclonal		#8146 (CST)	IB: 1:1000
Flag	Polyclonal		#F7425 (Sigma)	IB: 1:1000; IP: 4 μg
GATA-3	Monoclonal		M00593	IB: 1:2500; IP: 3~4 μg
GATA-3	Monoclonal		MA1-028	IB: 1:2000; IP: 4 μg
GATA-3	Monoclonal		#5852 (CST)	ChIP: 1:400; Immunohistochemistry: 1:100
Rabbit IgG	Polyclonal		#2729 (CST)	IP: same amount as the specific antibody
Mouse IgG	Monoclonal		#5415 (CST)	IP: same amount as the specific antibody
Rabbit IgG	Monoclonal		#66362 (CST)	ChIP: same amount as the specific antibody
GAPDH	Monoclonal		#2118 (CST)	IB: 1:1000
GFP	Monoclonal		#sc-9996 (Santa Cruz)	IB: 1:700; IP: 4 μg
GFP	Polyclonal		#NB600-308 (Novus Biologicals)	IB: 1:1000
p300	Polyclonal		#A300-358A	IB: 1:2000; IP: 3~4 μg
H3K9Ac	Monoclonal		#NB120-12179(Novus Biologicals)	IB: 1:1000
H3K27Ac	Monoclonal		#8173 (CST)	ChIP: 1:100
H3K4me1	Monoclonal		#5326 (CST)	ChIP: 1:50
H3K18Ac	Monoclonal		#13998 (CST)	IB: 1:1000
Histone H3	Monoclonal		#5192 (CST)	IB: 1:1000
TCF7	Monoclonal		#2203 (CST)	IB: 1:1000
ITK	Monoclonal		#2380 (CST)	IB: 1:1000
pITK (Y180)	Polyclonal		#5082 (CST)	IB: 1:1000
LAT-1	Polyclonal		#5347 (CST)	IB: 1:1000
PLC	Monoclonal		#5690 (CST)	IB: 1:1000
pPLC (Y783)	Polyclonal		#2821 (CST)	IB: 1:1000
RLK	Monoclonal		#H00007294-M01 (Abnova)	IB: 1:500
STAT5a	Monoclonal		#4807 (CST)	IB: 1:1000
ETS1	Monoclonal		#14069 (CST)	IB: 1:1000
NF-κB p65	Monoclonal		#8242 (CST)	IB: 1:1000
ZFPM1	Polyclonal		# A04565 (Boster)	IB: 1:500
ETS1	Monoclonal		#14069 (CST)	IB: 1:1000
STAT5a	Monoclonal		#4807 (CST)	IB: 1:1000
C-MYC	Monoclonal		#13987 (CST)	IB: 1:1000
p53	Monoclonal		#2527(CST)	IB:1:1000

CCR4	Monoclonal	PE	#561110 (BD Biosciences)	1ug/test
Mouse IgG	Monoclonal	PE	#555749 (BD Biosciences)	1ug/test
GATA-3	Monoclonal	APC	#48-9966-42 (ThermoFisher)	0.06ug/test
TCR Vβ 5.1,5.2	Monoclonal	FITC	#562087 (BD Biosciences)	0.5ug/test
Rat IgG	Monoclonal	APC	#48-4031-82 (ThermoFisher)	0.06ug/test
CD3e	Monoclonal	BV421	#562600 (BD Biosciences)	0.5ug/test
